# Seascape Genetics and Distinct Intraspecific Diversification of the Decapod *Nephrops norvegicus* in the Adriatic Sea

**DOI:** 10.1002/ece3.70358

**Published:** 2024-10-08

**Authors:** Marina Mašanović, Luka Žuvić, Iva Žužul, Igor Talijančić, Tanja Šegvić‐Bubić

**Affiliations:** ^1^ Faculty of Science Interdisciplinary Doctoral Study in Oceanology Zagreb Croatia; ^2^ Institute of Oceanography and Fisheries Split Croatia

**Keywords:** Central Mediterranean, decapods, gene flow, management, Norway lobster, population structure

## Abstract

Norway lobster *Nephrops norvegicus*, a prized decapod crustacean species, is found at different depths across the East Atlantic Ocean and Mediterranean Sea. Despite management efforts, the stocks are globally characterised as overexploited. In the present study, the impact of biogeographical boundaries on the phylogeographical and demographic population status was investigated within the Adriatic Sea, addressing important genetic indices for decapod functional conservation management. Central Mediterranean, Adriatic Sea A total of 482 individuals of *Nephrops* divided into the 12 samples were collected across biogeographical range of the Adriatic Sea. Using the mtDNA D‐loop and microsatellite markers, methods of phylogeography and seascape genetics were applied to infer offshore versus coastal population divergence, demography and structure. Significant findings include genetic differentiation between offshore and coastal samples, with higher diversity indices in open waters. The limited gene flow observed between these two areas emphasises the self‐sustained nature of coastal populations. Recent demographic changes in coastal populations reflect geographical constraints, fishing pressures and fluctuations in self‐recruitment success. Additionally, the study reveals historical biogeographic events shaping the Adriatic populations, with evidence suggesting lineage divergence during the upper Pleistocene and postglacial recolonisation from southern Adriatic refugia. The role of biogeographical conditions in shaping genetic structure and limited gene flow between inshore and offshore areas underscore the need for improved management strategies, emphasising the importance of marine protected areas in conserving coastal populations and maintaining overall genetic diversity of the Norway lobster in the Adriatic Sea. Genomic monitoring within current management practices is recommended.

## Graphical abstract

In the present study, the impact of biogeographical boundaries on the phylogeographical and demographic population status of *Nephrops norvegicus* was investigated within the Adriatic Sea, addressing important genetic indices for decapod functional conservation management.

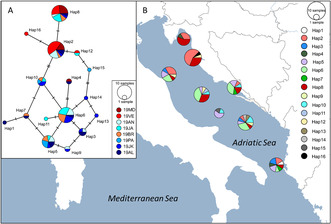

## 1 Introduction

The *Nephrops norvegicus* (hereafter referred to by genus alone) is a prized decapod crustacean species found at different depths across the east Atlantic Ocean and Mediterranean Sea (FAO 2023). They inhabit muddy sediments, where they build tunnels and express strong territorial behaviour (Aguzzi and Sardà 2008). Their emergence rhythm follows a strict diel and seasonal pattern and is influenced by several ecological and biological factors (Farmer 1974; Aguzzi, Company, and Sardà 2007; Aguzzi, Allué, and Sardà 2004; Naylor 2010). Like many other crustacean species, *Nephrops* undergoes a complex life cycle, including a planktonic larval stage lasting for 3–7 weeks depending on sea temperature and other environmental factors (Farmer 1975; Relini et al. 1998; Dickey‐Collas et al. 2000). During this stage, they are dispersed through the water column to distant locations, with successful settlement depending on factors such as prey presence, population density and sediment type (Johnson, Lordan, and Power 2013). Their growth is discontinuous, with moulting occurring more frequently in juveniles and at least once in a year in older individuals (Haynes et al. 2016). Variations in growth rates and size at sexual maturity can be found among *Nephrops* populations in the Mediterranean Sea and Atlantic Ocean and are discussed as the consequence of variance in environmental (temperature, sediment type, etc.) and biological factors (density, prey presence, etc.) (Johnson, Lordan, and Power 2013; Mytilineou et al. 1998; Powell and Eriksson 2013). These differences were also reported for the Adriatic *Nephrops* stock, with numerous studies indicating the presence of distinct subpopulations (Froglia and Gramitto 1981, 1988; Vrgoč et al. 2004; Angelini et al. 2020; Melaku Canu et al. 2021). Vrgoč et al. (2004) noticed slower growth rates and delayed sexual maturity in *Nephrops* from the channel areas of the northern Adriatic Sea, while Angelini et al. (2020) identified different subpopulations in the Adriatic Sea based on morphometric characteristics. However, allozyme characterisation has not revealed differences among Adriatic *Nephrops* from trawling grounds, suggesting environmental influences on growth and maturity (Mantovani and Scali 1992).

Predominantly fished by Italian and Croatian fleets (FAO 2023), the Adriatic *Nephrops* population is managed as a single stock unit, harvested with creels in channel areas and bottom trawls in both channel areas and the open sea (Brčić et al. 2018). Despite management efforts, FAO reports indicate overexploitation of the stock in the past decade (FAO 2020). Given the biological differences (in the size at the onset of maturity and growth) and the necessity for accurate stock assessment, the Scientific, Technical and Economic Commission for Fisheries of the European Commission recommended research into stock identification within geographical survey areas (GSA 17 and 18) (STECF 2015). In general, there is widespread acknowledgment that prolonged larval planktonic stages promote gene flow, leading to minimal differentiation among adjacent populations, even across extensive geographic areas (Macpherson and Raventós 2006). Despite the long larval planktonic stage of *Nephrops* (3–7 weeks), natural barriers formed by the Croatian islands can reduce the dispersal capabilities of *Nephrops* larvae, leading to isolation of certain subpopulations and preventing gene flow, which in turn results in differences between the subpopulations and increases their sensitivity to fishing activities. Recent research applying integrated oceanographic models has suggested the presence of at least three subpopulations in the Adriatic Sea (Melaku Canu et al. 2021). Their accurate identification holds significant implications for management of the Adriatic *Nephrops* fishery, with population genetic studies emerging as a reliable tool (Pavičić et al. 2020; Spedicato et al. 2022; Westgaard, Søvik, and Johansen 2023).

In the past decade, population genetic studies focusing on *Nephrops* inhabiting the Atlantic Ocean, Icelandic and Scottish waters and the Mediterranean Sea have provided new polymorphic markers that provide better insight into the population structure of *Nephrops* (Streiff et al. 2001, 2004; Stamatis et al. 2004; Skirnisdottir et al. 2010; Pampoulie et al. 2011; Gallagher et al. 2019). While most microsatellite studies have not revealed significant levels of population differentiation (Streiff et al. 2001, 2004; Skirnisdottir et al. 2010; Pampoulie et al. 2011), mitochondrial D‐loop DNA analyses have revealed genetic isolation between Atlantic and Mediterranean populations (Gallagher et al. 2019). Recently, the European Fisheries Commission initiated the MED_UNITs project, focusing on stock identification of key demersal species in the Mediterranean Sea, including *Nephrops*. For the first time, high‐quality single‐nucleotide polymorphisms were employed to discern potential genetic variations among the eastern Adriatic, and central and western Mediterranean *Nephrops* populations (Spedicato et al. 2022).

Although *Nephrops* has recently been the subject of several population genetic studies in the Mediterranean Sea, little is known about the *Nephrops* population dynamics in the Adriatic Sea and there is limited knowledge of the species genetic structure on a fine scale. Thus, in the present study we analysed the genetic structure of *Nephrops* from the Adriatic Sea using microsatellite loci and the mtDNA d‐loop region. The aims were to: (i) provide detailed information on the genetic structure of *Nephrops* from the Adriatic Sea; (ii) estimate genetic diversity and reconstruct its demographic history based on the mtDNA D‐loop marker and (iii) explore geographical partitioning of *Nephrops* inhabiting inshore and offshore areas, as a basis for understanding species population dynamic.

## 2 Materials and Methods

### 2.1 Tissue Sampling and DNA Extraction

**FIGURE 1:**
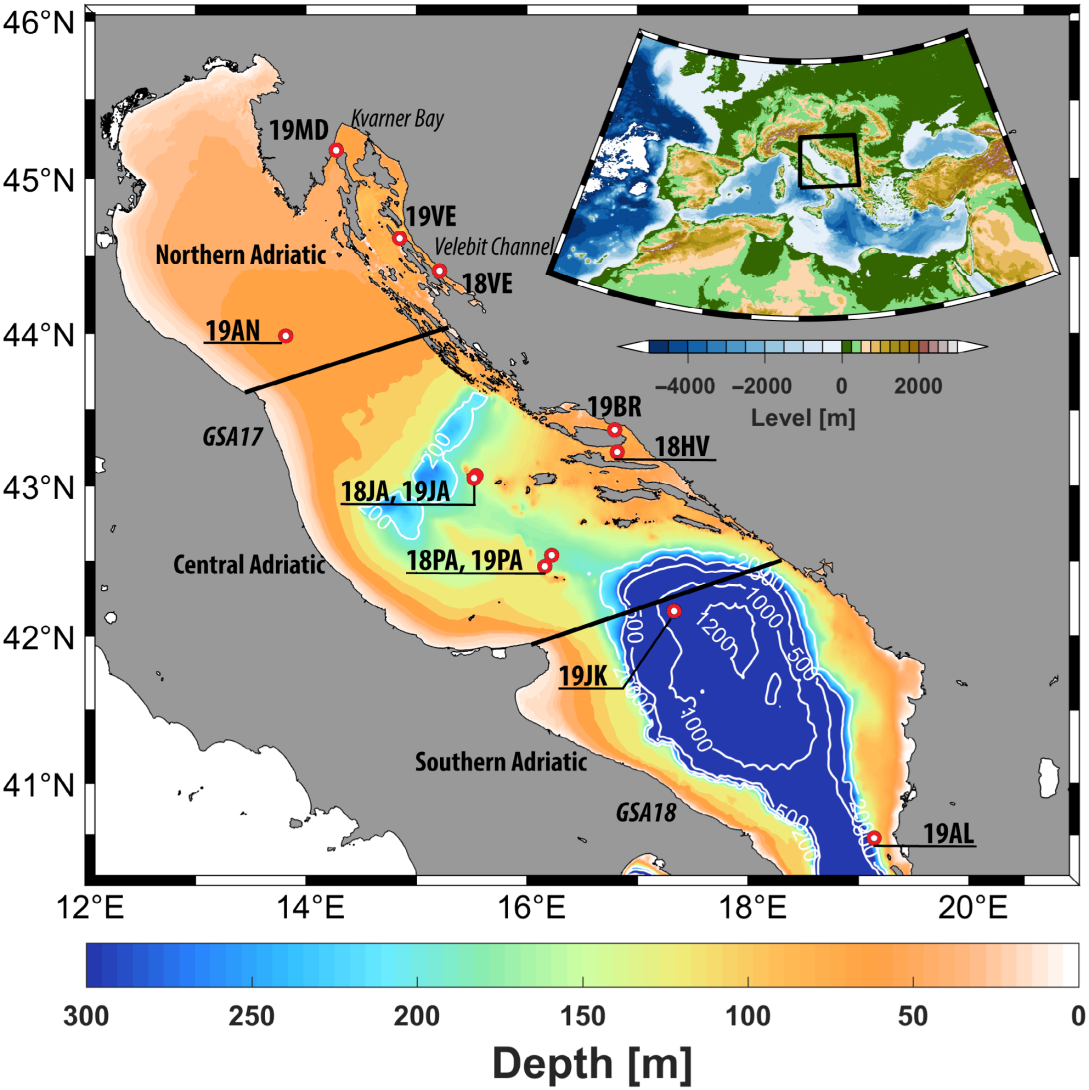
Adriatic Sea bathymetry with sampling locations of decapod *Nephrops norvegicus* where the first two letters of location abbreviations denote the sampling year (2018, 2019) and the second two letters denote the abbreviations for sampling sites (AL, Valona; AN, Ancona; BR, Brač channel; HV, Hvar channel; JA, Jabuka Pit; JK, South Adriatic Pit; MD, Mošćenička Draga; PA, Palagruža; VE, Velebit channel). More information about samples abbreviations and sampling years are provided in Table 1. The boundaries of the three Adriatic sub‐basins and FAO Geographical Subareas (GSAs) 17 and 18 are highlighted. Isobaths indicate depths of 250, 500, 1000 and 1200 m. The colours represent different depths. The figure was prepared in MATLAB 2014a (www.mathworks.com) and GIMP 2.8.16 (www.gimp.org) software.

A total of 482 individuals of *Nephrops* were collected from nine sampling sites in 2018 and 2019 (Table 1 and Figure 1). Collection included inshore and offshore sites in the three main biogeographical subregions of the Adriatic Sea (northern, central and southern). *Nephrops* from three locations (Velebit channel, Jabuka Pit and Palagruža) were sampled in two consecutive years, resulting in a total of 12 sample groups (hereafter referred as samples). Individuals were mainly collected from the landings of artisanal fisheries, with those from inshore locations caught using creels, while offshore individuals were collected using bottom trawlers. A tissue sample of the first pereiopod was collected from each captured individual and preserved in 96% ethanol, and sex and carapace length (mm) were measured. Depending on the location and year of sampling, sample sizes ranged from 24 to 60 individuals and included both juveniles and adults with carapace lengths ranging from 26 to 60 mm. Samples were coded according to the sampling year (18, 2018; 19, 2019) and sampling location.

**TABLE 1:** Information of sampling locations in the Adriatic Sea, year and code, along with the number of individuals of Norway lobster *Nephrops norvegicus* that were genetically assayed with 12 putatively neutral microsatellites (SSR) and mtDNA D‐loop marker.

Country	Site	Pop ID	No. samples		Sampling year	Latitude	Longitude
			SSR	mtDNA			
Inshore samples							
Croatia	Mošćenička Draga (Kvarner Bay)	19MD	30	11	2019	45.18056	14.27401
Croatia	Velebit channel	18VE	35		2018	44.40563	15.20894
Croatia	Velebit channel	19VE	60	16	2019	44.61681	14.84758
Croatia	Hvar channel	18HV	24		2018	43.22365	16.81698
Croatia	Brač channel	19BR	42	12	2019	43.36967	16.79404
Offshore samples							
Italy	Ancona	19AN	46	12	2019	43.98444	13.81667
Croatia	Jabuka Pit	18JA	35		2018	43.0676	15.5398
Croatia	Jabuka Pit	19JA	30	13	2019	43.0506	15.522
Croatia	Palagruža	18PA	46		2018	42.53909	16.22454
Croatia	Palagruža	19PA	55	7	2019	42.46493	16.16208
Croatia	South Adriatic Pit	19JK	41	13	2019	42.16609	17.33193
Albania	Valona	19AL	38	11	2019	40.62621	19.14195
Total			482	95			

### 2.2 Molecular Procedures

Total genomic DNA from individual pereiopod tissues was extracted by proteinase K digestion followed by phenol‐chloroform standard extraction protocol. Both the quality and quantity of DNA were determined spectrophotometrically (IMPLEN N50, Germany). A portion of the mitochondrial D‐loop (~280 bp) was amplified using specific PCR primers JG2 F 5′‐ CTA CAG ATT TCG TCT ATC AAC ‐3′ and NnD R 5′‐ GCT CTC ATA AAC GGG GTA TGA ‐3′ for *Nephrops* according to the protocol of Gallagher et al. (2019). Product sequencing was performed by Macrogen (Amsterdam, Netherlands) on an ABI 3730 automated sequencer. Due to poor product amplification, only 95 individuals from eight different locations were included in the following analyses.

For microsatellite analysis, a total of 482 individuals were successfully genotyped with 12 microsatellite markers developed for *Nephrops* and *Homarus gammarus* (André and Knutsen 2010; Streiff et al. 2001; Skirnisdottir et al. 2010; Table S1). These were divided into three PCR multiplexes and amplified using the Qiagen multiplex kit and standard primer dyes (FAM, NED, VIC and PET, Applied Biosystems). Amplification was performed in 12.5 mL reactions. The final concentrations of all primers were uniformly adjusted to 0.2 μM. PCR conditions were as follows: initial denaturation at 95°C for 5 min, 25 cycles at 95°C for 30 s, annealing at 57°C for 90 s and elongation at 72°C for 30 s, with final elongation at 60°C for 30 min. Fragments were separated on an ABI3130 automated sequencer with the 500 LIZ dye Size Standard (Applied Biosystems) using the services of Macrogen (Macrogen, Seoul, South Korea). Genotypes were analysed using GeneMapper software v.3.5 (Applied Biosystems).

### 2.3 Mitochondrial DNA

#### 2.3.1 Genetic Diversity, Structure and Evolutionary Analysis

Sequence alignment was performed in Mega7 using the ClustalW tool (Kumar et al. 2016). All mtDNA sequences of *Nephrops* were deposited in NCBI under accession numbers PP478077‐PP478092. Molecular diversity was measured using Dnasp 5.19 (Librado and Rozas 2009), calculating the number of haplotypes (*H*), polymorphic sites (*S*) and haplotype and nucleotide diversity. Pairwise genetic differentiation between populations using the fixation index *F*_ST_ and hierarchical analysis of molecular variance (AMOVA) at the inshore/offshore level was tested with 10,000 permutations in ARLEQUIN (Excoffier and Lischer 2010). Genetic relationships and haplotype divergence times were estimated using the Bayesian approach in BEAST2 v2.7.3 (Bouckaert et al. 2019). All haplotypes in the study were included along with *Homarus americanus* and *H. gammarus* haplotypes (GenBank Accession Numbers NC_015607.1, MH747083.1), which were used as outgroups. Details of calibration points and parameters from BEAST are provided in Appendix S1. To determine genealogical relationships of haplotypes, a median‐joining haplotype network was created using PopART v1.7 (Leigh and Bryant 2015). Finally, a neighbour‐net tree was built with SplitsTree4 (Huson and Bryant 2006) using the default parameters.

#### 2.3.2 Inference of Demographic History

Signatures of population demographic changes (bottlenecks or expansions) in *Nephrops* were first assessed using Tajima's *D* (Tajima 1989) and Fu's *F*_ST_ (Fu 1997) statistics in Arlequin v.3.5. (Excoffier and Lischer 2010). Second, demographic changes were analysed with a mismatch distribution using a spatial expansion model. The sum of squares of deviations (SSD), raggedness index (*r*) and associated *p* value were also calculated using Arlequin v.3.5. With a non‐significant SSD value, the population expansion hypothesis cannot be rejected, while a non‐significant Raggedness index indicates a good fit of the data to the spatial expansion model. The demographic expansion factor tau (*τ*) with a 95% confidence interval was also estimated, and the time at which the expansion event occurred was dated according to the formula *T* = *τ*/2 *μk*, where *T* is the estimated time in millions of years (My) since expansion, *μ* is the mutation rate per site per My, and *k* is the sequence length. Here, a mutation rate of 32% per 10^6^ years per site was tested. To analyse changes in relative population sizes over time, molecular‐based demographic fluctuation was estimated based on *Nephrops* haplotypes in BEAST v2.7.3 using the Bayesian skyline plot (BSP) method (Drummond et al. 2005). The mean clock rate parameter was used as previously estimated in the time calibration analysis. All demographic indices and BSP analyses were performed for the entire data set and for each clade derived from Bayesian phylogenetic analysis.

### 2.4 Microsatellite Data

#### 2.4.1 Genetic Diversity, Differentiation and Structuring

MICROCHECKER v.2.2.3 (Van Oosterhout et al. 2004) and FREENA (Chapuis and Estoup 2007) were used to assess the presence and frequency of null alleles for each locus. The linkage disequilibrium (LD), departures from Hardy–Weinberg equilibrium (HWE), observed and expected heterozygosity (*H*_e_, *H*_o_) and fixation index (*F*_IS_) were assessed using GENEPOP 4.0 (Rousset 2008). The Bonferroni correction was applied to adjust significance levels for multiple tests (Rice 1989). The mean number of alleles per locus (*A*) and the mean effective number of alleles across all loci (*A*_e_) were calculated using POPGENE 1.32 (Yeh, Yang, and Boyle 1999). Differences in heterozygosity and allelic richness (*A*_r_) between seascapes (i.e., inshore and offshore) were assessed using FSTAT 2.9.3 (Goudet 2022). The statistical power of genetic homogeneity was evaluated for all samples and markers using POWSIM 4.0 using Fisher's exact tests (Ryman and Palm 2006). Pairwise and global *F*_ST_ values were calculated using ARLEQUIN v.3.5 and applying 10,000 permutations. The genetic structure of *Nephrops* was analysed using the Bayesian clustering program STRUCTURE 2.3.4 (Pritchard, Stephens, and Donnelly 2000) with the following parameters: population admixture and a priori location (LOCPRIOR) with a burn‐in of 50,000 interactions followed by a run of 500,000 MCMC steps and *K* values from 1 to the maximum number of groups examined, each with 20 replicates. The most likely number of clusters was determined using the ln *P*(*D*) and delta *K* values implemented in Structure Harvester 0.6.93 (Earl and von Holdt 2012) and visualised using CLUMPP (Jakobsson and Rosenberg 2007) and DISTRUCT (Rosenberg 2004). In addition, discriminant analysis of principal components (DAPC), implemented in the Adegenet package (Jombart 2008) for R (R Core Team 2017), was applied using the dapc function and the xvalDapc function to optimise the retained number of principal components (PC).

#### 2.4.2 Demographic Changes, Effective Population Size and Migration Rate

To determine recent declines in effective population sizes (*N*_e_) over a relatively short period of several *N*_e_ generations, BOTTLENECK 1.2.02 (Piry, Luikart, and Cornuet 1999) was used with 20,000 replications, a two‐phase model (TPM) with 90% single‐step mutations and 10% variance among multiple steps. The probability of significant heterozygote excess was tested using a one‐tailed Wilcoxon signed‐rank test, as recommended by Piry, Luikart, and Cornuet (1999).

Both BayesAss1.3 (Wilson and Rannala 2003) and Migrate‐n software (Beerli and Felsenstein 1999; Beerli 2006) were used to examine contemporary migration based on a few generations and historical migration (on the order of thousands of years ago) followed by divergence across a genetic discontinuity between populations from open seas and channels, which was also identified by mtDNA sequence data. For BayesAss, the developer's recommendations regarding parameter settings were followed and each MCMC run included 3 × 10^6^ iterations, with the first 10^6^ iterations discarded as burn‐in. The log‐probability of each run was analysed using TRACER v.171 (Rambaut et al. 2018).

Different models of gene flow between inshore and offshore seascapes were evaluated and compared using a Bayesian structured coalescent approach implemented in Migrate‐n v.5.0 on the CIPRES portal (Miller, Pfeiffer, and Schwartz 2010), estimating mutation‐scaled population sizes (Theta), immigration rates (*M*) and divergence times (*D*). The Brownian motion mutation model and constant mutation rates for all loci were used as initial parameters. After testing the parameters in the initial runs, the uniform prior distributions (min, max, delta) were set as follows: Theta = 0.0, 100.0, 10.0; *M* = 0.0, 100, 10.0; *D* = 0.0, 60.0, 6.0. Each model was run using the metropolis‐coupled MCMC and a static heating scheme (default parameters). Ten heated replicate chains were used for each locus, and each replicate was run for 1 × 10^6^ steps, with 1 × 10^5^ samples recorded every 10 steps and the first 1 × 10^5^ steps discarded as burn‐in. Model selection was performed by calculating Bayes factors from marginal likelihoods as described in Beerli and Palczewski (2010). Several demographic models were tested, ranging from the simplest models, such as a panmictic, unidirectional and bidirectional models, to more complex models that include divergence events with and without immigration (Figure S1.1). Because the model with the inshore samples split from the offshore samples without immigration was initially the best fit, an additional model was tested using an ancient unsampled population (‘ghost’ population) as the ancestor of the inshore and offshore samples enabling independent splitting from one another (as suggested by P. Beerli, pers. comm.; Figure S1.1). To convert the divergence (posterior distribution) to the number of generations since the coalescence of populations, the posterior distribution was divided by the expected mutation rate. Later, the time of divergence to years before the present was estimated using a generation time of three years (Relini et al. 1998). The mutation rate of 0.00045 per locus per generation was considered, as a classical average value derived from many different species (Whittaker et al. 2003; Macedo et al. 2019).

Finally, the NeEstimator V2 program (Do et al. 2014), which implements the linkage disequilibrium method (Waples and Do 2008) for a single sample, was used to estimate the contemporary effective population size for all populations and seascapes. Alleles with frequencies below 0.02 were removed from the analysis to balance precision with the potential bias of highly polymorphic microsatellite loci.

## 3 Results

### 3.1 Mitochondrial DNA Diversity, Phylogeography and Population Structure

**FIGURE 2:**
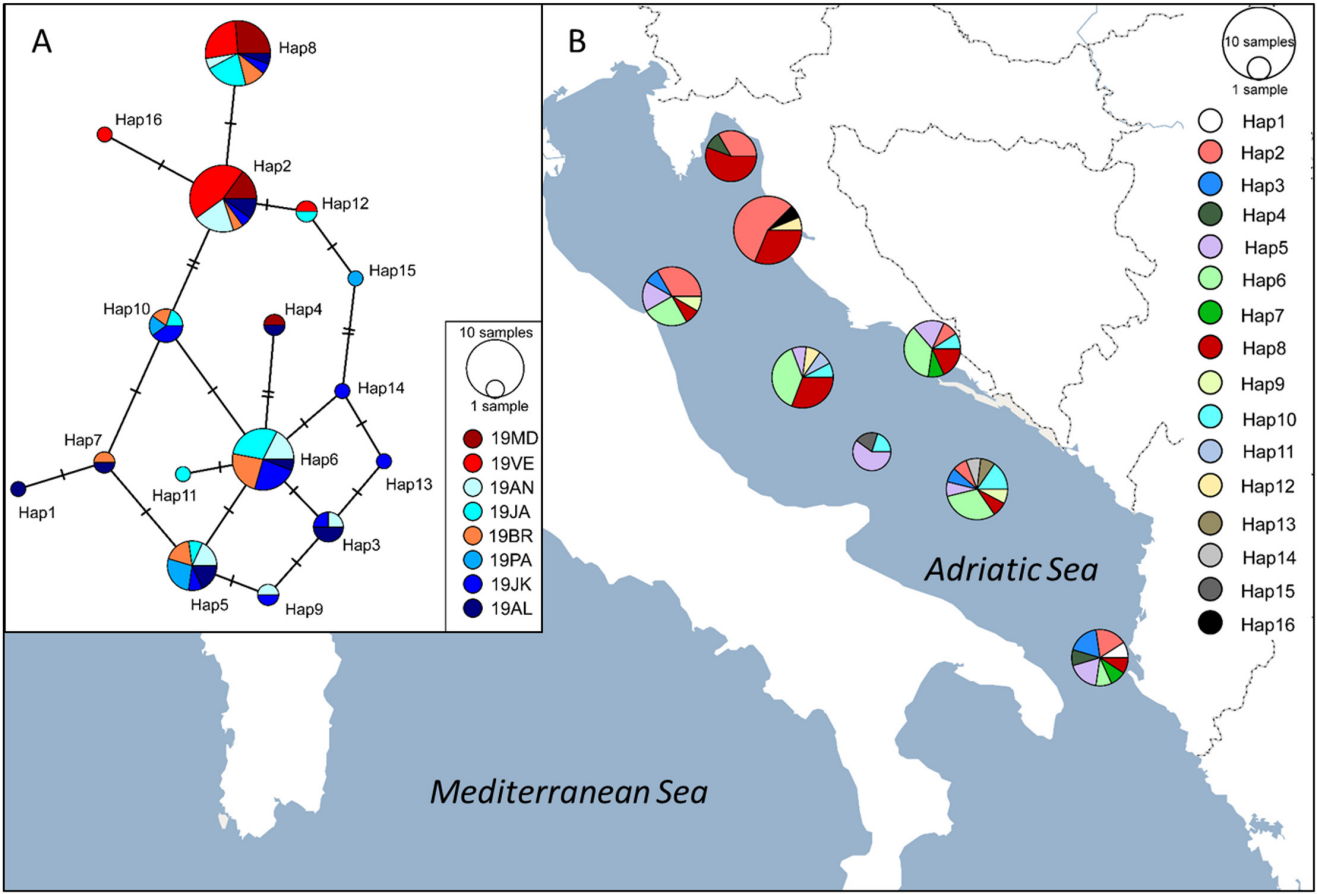
Median‐joining network and geographic distribution of *Nephrops norvegicus* haplotypes in the Adriatic Sea. (A) Network of 95 D‐loop sequences. Coloured circles represent haplotypes found in sampled populations whose sizes are proportional to the number of individuals. Number of mutation steps is shown as hatch marks. (B) Geographic distributions of the mitochondrial haplotypes. The pie charts on the map display the haplotype frequencies found at each location. Samples codes are given in Table 1.

**FIGURE 3:**
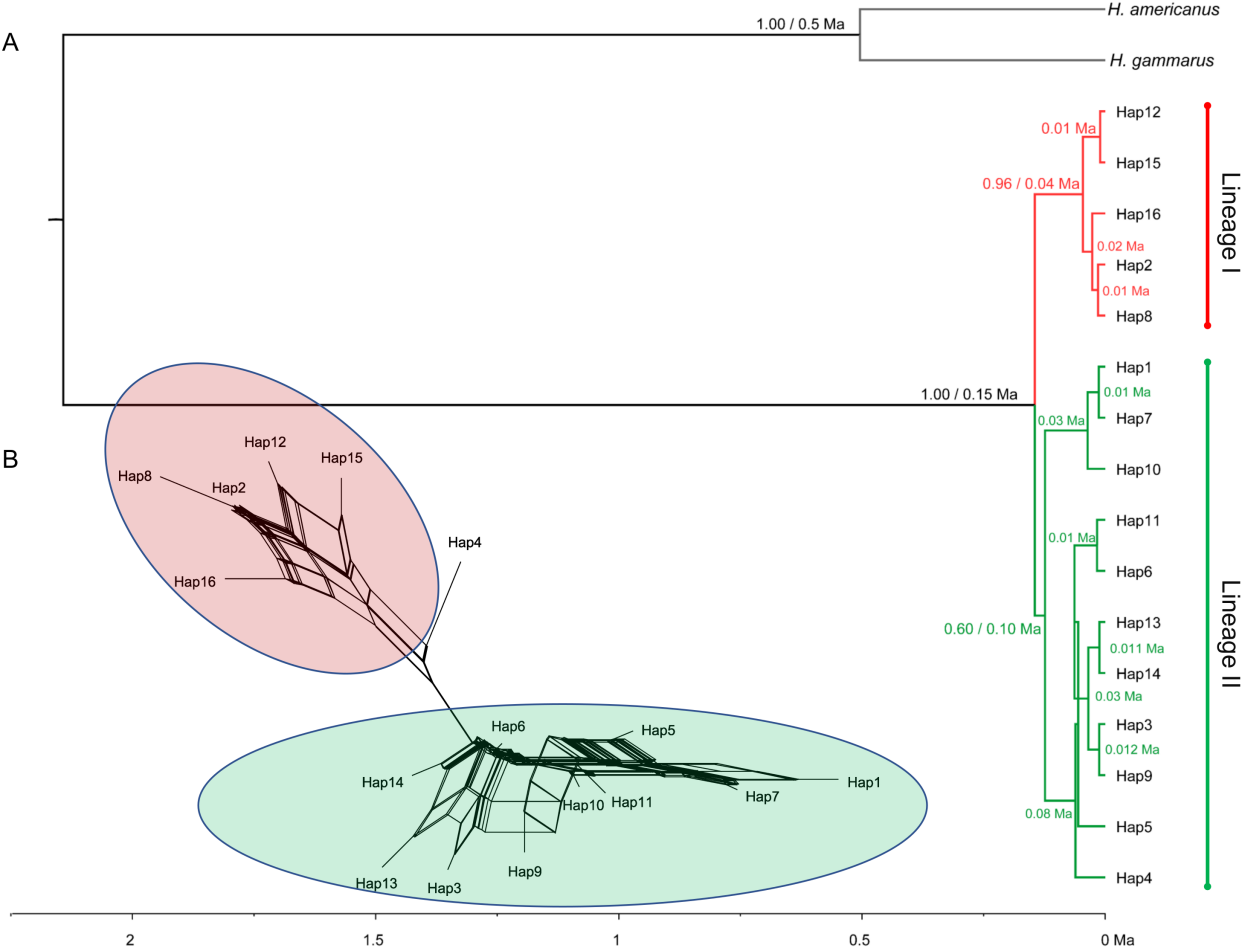
Genetic structure of *N. norvegicus*. (A) MCC tree from BEAST analysis based on the mtDNA dataset of 16 haplotypes. Values above the branches of the tree indicate (i) posterior probability values greater than 0.5 and (ii) estimates of divergence times (million years, Ma). *H. americanus* and *H. gammarus* were used as outgroups. The scale bar shows the time in millions of years from the present day. Lineages I and II are indicated by red and green lines, respectively. (B) A neighbour‐net tree constructed from the mtDNA dataset with 31 haplotypes using SplitsTree4, where the red and green circles represent haplotypes from lineage I and lineage II, respectively.

Amplification of *Nephrops* genomic DNA with the JG2 and NnD primers yielded a PCR product of ~550 bp, including the hypervariable fragment of the D‐loop of ~280 bp detected by Gallagher et al. (2019). The recently recognised D‐loop fragment was obtained from unambiguously aligned sequences, with 95 of the total 132 sequenced samples passing quality assessment and resolving 16 haplotypes (GenBank accession numbers PP478077‐PP478092). The genealogical relationships of the haplotypes revealed two well‐supported *Nephrops* lineages with an estimated genetic distance of *D* = 0.02 (Figures 2A and 3). All sampled individuals from the northern coastal waters (19MD, 19VE) and some from the middle coastal waters (19BR—37%) of the eastern Adriatic were assigned to lineage I, while lineage II consisted mainly of individuals sampled in the offshore waters (83%) of the Adriatic Sea. Regarding the distribution of haplotypes between lineages, the most common haplotypes (Hap2 and Hap8) were assigned to lineage I and occurred in the majority of sampled groups along the Adriatic Sea, dominating the abundance in samples from inshore waters (~65%, Figure 2B). The haplotypes of lineage II, with the most frequent variants Hap5 and Hap6, were widely distributed throughout the Adriatic Sea, except in northern inshore waters. Only Hap4 was found in both marine areas. A total of six haplotypes were identified as private haplotypes, five of which were from offshore areas. The overall diversity of haplotypes and nucleotides was 0.86 and 0.01, respectively (Table 2). The southern offshore samples (19JK, 19AL) had the highest values for both indices compared to the remaining ones. Consequently, lineage II had higher index values than lineage I (Table 2).

**TABLE 2:** Genetic diversity, neutrality test statistics and mismatch distribution analysis for the Norway lobster *Nephrops norvegicus* collection from the Adriatic Sea based on the mtDNA gene. Samples codes are as in Table 1.

Samples	Genetic diversity estimates				Neutrality tests		Spatial expansion parameters		
	*H*	*S*	*H*_d_	*π*	Tajima's *D*	Fu's *F*_s_	*τ* (SD_95%_)	SSD	*r*
Inshore									
19MD	3	4	0.63 ± 0.12	0.005 ± 0.00	−0.689	0.735	0.46 (0.0–4.55)	0.049	0.21
19VE	4	3	0.62 ± 0.09	0.003 ± 0.00	−0.627	−1.018	0.72 (0.10–51.83)	0.063*	0.21
19BR	6	5	0.86 ± 0.08	0.009 ± 0.01	1.065	−1.331	0.76 (0.42–124.1)	0.027	0.09
Offshore									
19AN	6	6	0.85 ± 0.07	0.009 ± 0.01	0.927	−0.785	2.10 (1.19–7.34)	0.031	0.07
19JA	6	7	0.80 ± 0.08	0.009 ± 0.01	0.389	−0.569	3.24 (1.23–10.47)	0.049	0.13
19PA	3	5	0.70 ± 0.22	0.008 ± 0.01	−0.562	0.803	3.73 (0.0–168.0)	0.125	0.59
19JK	9	7	0.91 ± 0.06	0.009 ± 0.01	−0.214	−4.982**	1.62 (0.62–3.61)	0.008	0.07
19AL	8	8	0.95 ± 0.05	0.011 ± 0.01	0.214	−3.140*	3.45 (1.43–4.93)	0.016	0.07
Lineage I	6	6	0.62 ± 0.04	0.003 ± 0.01	−0.989	−1.646	0.88 (0.28–1.60)	0.017*	0.16*
Lineage II	11	7	0.81 ± 0.04	0.005 ± 0.01	−0.390	−5.093**	1.48 (0.92–2.22)	0.009	0.10
Total	16	9	0.86 ± 0.02	0.010 ± 0.01	1.036	−4.230**	1.99 (0.63–46.78)	0.043	0.18

*Note:* Lineages were previously defined by the mitochondrial phylogenetic tree. Significant values are indicated with asterisks, *p < 0.05 and **p < 0.01.

Abbreviations: *H*, haplotypes; *H*_d_, haplotype diversity; *r*, Harpending's Raggedness Index; *S*, polymorphic sites; SSD, Sum of Squared Deviations; *π*, nucleotide diversity; *τ*, expansion parameter.

A moderate and significant overall *F*_ST_ (0.135) was found among the samples of *Nephrops*. Pairwise *F*_ST_ values ranged from −0.008 to 0.35, with significant heterogeneity observed when comparing samples from northern inshore waters (19VE, 19MD) to any sampling site in the Adriatic Sea (*p* < 0.05, Table S1.2), with the exception of 19MD and the neighbouring location 19AN (*p* > 0.05). A test of the hypothesis that genetic variation is structured at the inshore/offshore level showed that only 3.84% of the total variance was distributed between seascapes. When 19BR was excluded from the inshore group, the total variance between groups was 12.5% (*F*_CT_, *p* < 0.05), with the majority of genetic variation was found within populations (*F*_ST_ = 84.5%, *p* < 0.001).

### 3.2 Divergence Dating and Demographic Statistics Based on Mitochondrial Gene Sequences

The Bayesian tree constructed using BEAST places the time of initial divergence of the two major lineages in the upper Pleistocene (Figure 3, 0.15 Ma) with intense haplotype diversification within each lineage in the Holocene, that is, after the Last Glacial Maximum (LGM; ~0.01 Ma). Significantly negative values for Fu's *F*_s_ were observed only for the two southernmost offshore samples (19JK, 19AL) from the Adriatic and consequently for lineage II (Table 2). SSD and Raggedness index values supported a sudden expansion model for the southern samples and lineage II. Due to non‐significant values for Fu's Fs and significant SSD and Raggedness index values, the expansion hypothesis was rejected for lineage I (Table 2). Bayesian Skyline Plot (BSP) analysis supported the recent demographic expansion of lineage II and population stability of lineage I from marine transgression in the mid‐late Holocene (~5KY BP, Figure S2). The expansion of lineage II began slowly after lineage separation and continued into the recent past. In contrast, lineage I did not show pronounced demographic changes for a long time and only recently showed a change toward population reduction. According to the *τ*‐value of 1.48 (lineage II) (Table 2), the calculated expansion time for *Nephrops* in the Adriatic waters was 9250 (95% CI of 5750 to 13,870) years ago, supporting the results of the BSP analysis.

### 3.3 Microsatellite Markers of Genetic Diversity and Population Structure

A total of 482 individuals of *Nephrops* were genotyped at 12 microsatellite loci (Table 1, Figure 1 and Table S1.1). MICROCHECKER detected a high frequency (> 15%) of null alleles for the PLH33 and Nnmic1‐C12 loci, so these loci were excluded from further analyses. Following exclusion of the loci, no support was detected for linkage disequilibrium between the locus pairs, with no significant deviations from Hardy–Weinberg equilibrium, as revealed by Fisher's exact test (Table S1.3). In addition, *F*_ST_ values calculated with and without the use of the Excluding Null Alleles correction method were comparable (0.0096 vs. 0.0098) with overlapping 95% CI (0.0049–0.0146).

All loci were polymorphic, with the number of alleles per locus ranging from 6 to 34 (Table S1.3). The average number of alleles was approximately 15% lower in the inshore than in the offshore samples. The observed heterozygosity (*H*_o_) showed different degrees of genetic diversity (0.79–0.87) among samples, but the expected heterozygosity (*H*_e_) was less variable (0.86–0.88) and was significantly lower in the inshore than the offshore group (Table 3). Also, the indices of effective number of alleles (*A*_e_) and allelic richness (*A*_r_) were reduced in the inshore in contrast to offshore samples.

**TABLE 3:** Summary statistics for genetic variation of Norway lobster *Nephrops norvegicus*, showing the average number of alleles (*A*), effective number of alleles (*A*_e_), allelic richness (*A*_r_), expected (*H*_e_) and observed (*H*_o_) heterozygosity, fixation index (*F*_IS_) and effective population size (*N*_e_) for 10 microsatellite loci. Wilcoxon test *p* value for heterozygote excess compared to expectations at mutation‐drift equilibrium (*P*_wil_) for each sample is also presented.

Samples	*A*	*A*_e_	*A*_r_	*H*_o_	*H*_e_	*F*_IS_	*P*_wil_	*N*_E_
Inshore								
19MD	14.5 ± 7.3	9.5 ± 5.6	12.6 ± 5.6	0.83 ± 0.1	0.87 ± 0.1	0.052	**0.001**	386 (125, ∞)
18VE	14.4 ± 6.1	9.1 ± 5.9	12.1 ± 5.1	0.84 ± 0.2	0.85 ± 0.1	0.011	0.161	193 (109, 683)
19VE	17.4 ± 8.0	10.4 ± 6.7	12.6 ± 5.2	0.79 ± 0.1	0.87 ± 0.1	0.084*	**0.045**	9715 (649, ∞)
18HV	14.1 ± 6.7	9.4 ± 5.5	13.0 ± 5.9	0.87 ± 0.1	086 ± 0.1	0.012	**0.007**	∞ (3869, ∞)
19BR	16.0 ± 8.4	9.7 ± 6.8	12.4 ± 5.8	0.84 ± 0.1	0.86 ± 0.1	0.026	**0.005**	186 (115, 435)
Total	23.3 ± 11.5	11.8 ± 8.3	12.5 ± 5.7^a^	0.83 ± 0.1^a^	0.86 ± 0.1^a^	0.042^a^		553 (401, 860)
Offshore								
19AN	18.7 ± 9.7	11.2 ± 7.5	13.0 ± 6.4	0.79 ± 0.1	0.88 ± 0.1	0.103*	0.053	∞ (670, ∞)
18JA	16.6 ± 9.0	9.7 ± 7.0	13.1 ± 5.6	0.83 ± 0.1	0.86 ± 0.1	0.041	0.348	344 (142, ∞)
19JA	15.5 ± 7.2	9.8 ± 5.4	13.6 ± 5.7	0.79 ± 0.2	0.88 ± 0.1	0.105*	0.080	980 (152, ∞)
18PA	18.5 ± 9.1	10.7 ± 6.8	13.6 ± 6.5	0.81 ± 0.2	0.87 ± 0.1	0.071*	0.246	435 (200, ∞)
19PA	19.7 ± 10.5	11.7 ± 8.1	13.6 ± 5.9	0.83 ± 0.1	0.88 ± 0.1	0.064*	0.097	4611 (389, ∞)
19JK	17.6 ± 8.4	10.9 ± 7.6	13.8 ± 6.1	0.85 ± 0.1	0.87 ± 0.1	0.029	0.080	2252 (301, ∞)
19AL	17.6 ± 8.6	10.8 ± 6.9	13.5 ± 6.1	0.80 ± 0.1	0.88 ± 0.1	0.097*	0.138	1417 (321, ∞)
Total	27.4 ± 14.3	13.1 ± 9.7	13.5 ± 6.1^b^	0.82 ± 0.1^a^	0.88 ± 0.1^b^	0.072^a^		26,930 (3128, ∞)
Total data set	29 ± 14.7		13.5 ± 6.0	0.82 ± 0.1	0.88 ± 0.1	0.069		2683 (1622, 7054)

* HWE population deviation and *F*_IS_ at *p* < 0.0003; Wilcoxon test *p* < 0.05 in bold; the superscript without same letters indicates statistical significance at *p* < 0.05 between sample groups.

The inbreeding coefficient, *F*_IS_, ranged from 0.01 to 0.10 in the data set and was significantly higher than zero in 50% of the samples (Table 3).

**FIGURE 4:**
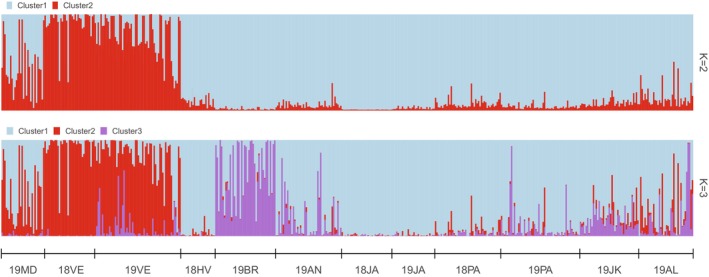
Bayesian clustering of *Nephrops norvegicus* inferred from STRUCTURE analysis of 10 loci dataset across 12 sample sites, where three clusters (*K* = 3) were assumed. For comparison, the analysis for *K* = 2 is also presented. See Table 1 for additional information on sample names.

POWSIM analysis revealed that the present microsatellite datasets could detect genetic differentiation up to *F*_ST_ = 0.0025 with 100% confidence and up to *F*_ST_ = 0.0012 with 92% confidence (*χ* ^2^, Fisher test). Global genetic differentiation across all 12 samples was 0.010 (*p* < 0.0001), demonstrating a relatively low to moderate level of differentiation among *Nephrops* collection. Within‐sample differentiation was twice as high for inshore than offshore samples (*F*_ST_: 0.014 vs. 0.007). After Bonferroni correction, 26 of 66 pairwise *F*_ST_ comparisons were statistically significant when permuted with Fisher's exact test (Table S1.4). High and significant pairwise interactions were observed between the inshore samples of the northern and central eastern Adriatic, but also between the inshore (18VE, 19VE and 19BR) and the majority of the offshore samples. Moreover, no gene flow interruptions were detected within the offshore samples, except for the Palagruza pit (18PA) sample, which showed significant interactions with all sampled groups (Table S1.4). It should be noted that in next sampling year, this pattern was not recorded for the Palagruza pit (19PA) sample. Based on the mean *L*(*K*) and delta *K* statistics, three discrete genetic clusters of *Nephrops* were derived using Bayesian cluster analysis (Figure 4, Figure S1.3). Inshore samples were assigned to all three clusters, of which two clusters were exclusively associated with the coastal seascape. The samples from the Velebit Channel from 2018 and 2019 (18VE and 19VE) formed the red cluster (Cluster2), while the sample from the Brač Channel (19BR) formed the purple cluster (Cluster3). The blue cluster was shared by all offshore samples and the inshore sample from the Hvar Channel (Cluster1). The northernmost sample from Kvarner Bay (19MD) showed a transitional pattern from nearshore to offshore origin, as 26% of individuals were assigned to Cluster2 and 53% to Cluster1, with an assignment score above 0.7 as the threshold for assigning individuals to nearshore or offshore origin (Figure 4). Interestingly, the assignment pattern at *K* = 2 remained the same as at *K* = 3, except for the Brač Channel (19BR) sample, which was assigned to the blue Cluster 1, shared by all offshore samples and the inshore sample from the Hvar Channel.

A hierarchical STRUCTURE analysis aimed at exploring additional population structure within the offshore samples (Cluster1 at *K* = 3) indicated pandemic, as the most likely number of populations was identified as *K* = 1 using the mean *L*(*K*) (data not shown). The presence of three clusters was also supported by the optimal number of clusters identified using DAPC and successive *K*‐means clustering analyses. The DAPC plot clustered groups similarly to the structure pattern in STRUCTURE, when each location was treated as an a priori cluster (Figure S1.4).

### 3.4 Demographic Pattern and Gene Flow Based on Microsatellite Data

High disparity of contemporaneous effective population sizes (*N*_e_) with respect to seascape origin was recorded (Table 3). On average, the minimum CI estimate of *N*_e_ was 7‐fold smaller in the inshore group (401) than in the offshore group (3128). In contrast to the offshore samples, the bottleneck test showed statistical evidence that *Nephrops* from inshore areas have experienced a recent reduction in population size, as Wilcoxon signed‐rank tests detected significant heterozygote excess (*p* < 0.05) under the two‐phase model in 4 of 5 inshore samples, respectively (Table 3).

Estimates of contemporary migration using the inshore‐offshore gene flow design revealed that samples from offshore areas were the source of exogenous allelic variants, with an estimated emigration rate of 0.32 toward neighbouring inshore areas. Very little gene flow was detected in the opposite direction (0.007), suggesting that the samples from inshore function as sink populations. Analysis of historical gene flow in Migrate‐n showed that the best model specifies an inshore split off from the offshore population without immigration, based on the likelihood of different migration models (Table 4, Figure S1). Considering the ‘ghost’ population as ancestral to inshore and offshore groups, the best model was the one describing the split of the ancestral population into the present inshore and offshore populations without ongoing immigration (Table 4). According to the best supported model, the mutation‐scaled offshore sample size was 35% larger than the inshore sample size, while the mutation‐scaled divergence time of both contemporary populations from the ancestral population overlapped (1.02–1.14; Table S1.5). In estimating the timing of colonisation, both contemporary populations descended from the ancestral one some 7650–8550 years ago, with an average microsatellite mutation rate of 0.00045 and a generation time of 3 years.

**TABLE 4:** Log marginal likelihoods and probabilities of biogeographic models for Norway lobster *Nephrops norvegicus* in the eastern Adriatic Sea. Two model designs were defined to test inshore/offshore lineage diversification and population connectivity in the Migrate‐n software: (a) two populations grouped as inshore/offshore and (b) introduction of an unsampled population (‘ghost’ population) as ancestral to the inshore and offshore groups.

Model description		Log (mL)	LBF	Model prob
(a) Inshore (INS) vs. offshore (OFF)				
1	Panmixia	−42699.4	−12031.9	0
2	Symmetric immigration	−41894.5	−11227.0	0
3	Immigration to the OFF	−37649.1	−6981.7	0
4	Immigration to the INS	−35604.7	−4937.3	0
5	Split OFF from INS without migration	−34289.9	−3622.4	0
6	Split OFF from INS with migration to OFF	−36557.8	−5890.3	0
7	Split INS from OFF without migration	−30667.4	0	1
8	Split INS from OFF with migration to INS	−39025.3	−8357.8	0
(b) Ancestral (ANC) vs. Inshore (INS) vs. offshore (OFF)				
9	Split INS and OFF from ANC lineage	−24494.5	0	1
10	Split INS and OFF from ANC lineage with symmetric immigration	−24964.0	−469.5	0
11	Split INS and OFF from ANC lineage with immigration to the OFF	−35283.2	−10788.6	0
12	Split INS and OFF from ANC lineage with immigration to the INS	−31037.2	−6542.7	0

Abbreviations: LBF, ln Bayes factor against the best model (7 and 9); Log(mL), Bezier approximation score.

## 4 Discussion

The fine‐scale population structure of *Nephrops* from the Adriatic Sea, as determined by both nuclear and mitochondrial markers, is presented for the first time. Using a total of 482 samples of *Nephrops* collected from two geographical subareas in the Adriatic (GSA17 and GSA18; FAO zone 37), the research was primarily focused on contrasting offshore and inshore samples across different seascapes, acknowledging the highly diverse coastline of the eastern Adriatic. Additionally, the study addressed overall population diversity, phylogeography and demographic patterns of *Nephrops*, that is, key parameters for sustainable and ecosystem‐based fisheries management. This is particularly important knowing that *Nephrops* stocks are globally characterised as overexploited (European Commission, Joint Research Centre, Scientific, Technical and Economic Committee for Fisheries (STECF) 2023) and is one of the most important crustacean species, ranking first by value and second by weight among the exploited crustaceans in the Mediterranean (FAO 2016).

Several significant findings have emerged from the present study. First, the examination of genetic distribution within the Adriatic basin has revealed a population differentiation of *Nephrops* and geographical partitioning according to both mtDNA and nuclear markers among individuals inhabiting open and inshore waters. Second, both markers showed higher levels of diversity indices in samples from open waters compared to those inhabiting inshore waters, including channels. This is supported by greater allelic richness, expected heterozygosity, haplotypes and nucleotide diversity. Additionally, the recent reduction in population size and decreased contemporary *N*_e_ values found in samples inhabiting inshore waters likely reflects recent demographic changes due to a combination of geographical and bathymetric constraints (Novosel et al. 2002), high fishing pressures (STECF 2020, 2022) and fluctuations in self‐recruitment success of these isolated populations (Melaku Canu et al. 2021; Sponaugle et al. 2002). Lastly, consistent with the geological events of the Holocene, notably after the Last Glacial Maximum (LGM; ~0.01 Ma), when sea levels reached their modern high levels with the northward intrusion of Levantine Intermediate Water on the slope (Piva et al. 2008), the colonisation of *Nephrops* followed by population expansion in the Adriatic Sea has been estimated using both molecular markers.

Only a few studies have investigated the population genetics of *Nephrops* within the Mediterranean Sea using various methods, such as allozymes, mtDNA RFLP and mtDNA CR. Both allozyme and mtDNA RFLP data (Maltagliati et al. 1998; Stamatis et al. 2004, 2006) have revealed low to moderate genetic differentiation between geographical regions (Atlantic vs. Mediterranean). However, no clear geographical pattern of genetic differentiation within the Mediterranean has been identified. The microsatellites applied here have previously only been applied for analysing populations inhabiting Icelandic and Norwegian waters (Pampoulie et al. 2011; Westgaard, Søvik, and Johansen 2023) and have never been used in the Mediterranean Sea.

Recently, through the Framework Contract for Scientific Advice for Fisheries in Mediterranean and Black Sea Waters (EASME/EMFF/2016/032), the EU Commission funded a study aimed at advancing fisheries assessment and management in the Mediterranean aligning biological and management units of priority species. Consequently, a comprehensive genetic study using 730 high‐quality SNPs significantly increased our knowledge related to stock boundaries, connectivity patterns and population genetic structure of *Nephrops* populations in the Mediterranean (Spedicato et al. 2022). For the first time, a significant differentiation of *Nephrops* from the eastern Adriatic Sea (GSA17) against those from the central and Western Mediterranean has been recorded, with high and significant genetic distances observed between the Adriatic Sea (GSA17 to 19) and the neighbouring basins in the western (GSA1 to 11) and eastern (GSA22) Mediterranean (Spedicato et al. 2022).

It appears that the semi‐enclosed Adriatic Sea represents a defined phylogeographic region within the Mediterranean (Patarnello, Volckaert, and Castilho 2007). Reduced gene flow has also been documented for the decapod *Homarus gammarus* (Pavičić et al. 2020) and other marine species (*Ruditapes decussatus*, Cordero, Peña, and Saavedra 2014; *Sepia officinalis*, Pérez‐Losada et al. 2007; *Solea solea*, Sabatini et al. 2018; *Paracentrotus lividus*, Maltagliati et al. 2010) between the Adriatic Sea and the broader Mediterranean. Nevertheless, a fine‐scale population study for *Nephrops* within the Adriatic Sea is still lacking.

### 4.1 Historical Biogeography and Demography

The present study gives the first insight into the clear allopatric lineage divergence with a geographical structure in the *Nephrops* population within the Adriatic Sea. Lineage I was supported with individuals from northern coastal waters (19MD, 19VE) and partially from middle coastal waters, while lineage II supported individuals from offshore waters. The time‐calibrated phylogeny suggested that the split of the two major lineages occurred during the upper Pleistocene, with intense haplotype diversification within each lineage in the Holocene, that is, after the LGM.

The Pleistocene epoch, characterised by cycles of glacial and interglacial periods, brought significant climatic fluctuations and sea level changes that shaped the Adriatic's marine biodiversity (Emig and Geistdoerfer 2004; Trincardi, Correggiari, and Roveri 1996). During the Riss‐Wurm interglacial phase (135,000–120,000 yBP), sea levels in the Adriatic were similar to current levels (Benac and Juračić 1998), allowing gene flow among lineages. However, as sea levels gradually declined over the subsequent 90,000 years, populations in the Velebit Channel and Kvarner Bay likely became isolated from those in the Middle Adriatic. The unique paleorelief of the Velebit Channel, with its steep and deep muddy bottoms and sporadic extreme submarine depressions, enabled these populations to persist even during the Wurm glaciation when sea levels were 100 m below present levels (Novosel et al. 2002).

The rapid sea level rise between 17,000 and 6000 yBP submerged much of the northern and central Adriatic, creating hydrographic and sedimentary conditions similar to the present. This likely contributed to the observed haplotype diversification within Adriatic lineages, particularly in populations from open‐sea areas, as evidenced by signs of sudden population expansion. While many species that entered the Mediterranean during alternating cold and warm periods became extinct when climatic conditions reversed, *Nephrops*, a boreal species that generally inhabits deeper regions in the Mediterranean compared to the North Atlantic, likely found refuge during glacial phases in the South Adriatic Pit, where depths reach up to 1270 m (Bianchi et al. 2012). Additionally, the southernmost Adriatic samples (19JK, 19AL) exhibit higher genetic diversity then middle Adriatic ones (19AN, 19JA, 19PA), suggesting the long‐term persistence of large populations, likely due to stable refugia in deeper waters. The postglacial recolonisation of the Adriatic from southern refugia northward is further supported by the south–north orientation of the basin and general counter‐clockwise circulation pattern, with currents flowing northerly along the eastern coast and returning southerly along the western coast (Orlić, Gačić, and LaViolette 2012).

Similarly, deep reefs have been identified as climatic refugia for the genetic diversity of *Saccorhiza polyschides*, a large habitat‐structuring brown alga, which allowed for the persistence of the largest genetic diversity pools of the species' distribution (Assis et al. 2016).

Despite the longstanding debate regarding the role of glacial refugia in shaping contemporary species distributions, our results suggest that lineage diversity has been predominantly shaped by drift and divergent selection in isolation of the northern channel clade, followed by secondary contact. Reproductive isolating mechanisms appear strong and sufficient to prevent introgression, underscoring the complex interplay between historical biogeography and demographic processes in the evolutionary history of the *Nephrops* population in the Adriatic Sea.

Though the mtDNA and microsatellite markers used in the present study showed consistency in terms of population diversities, we argue that biogeographical inferences based solely on hypervariable mtDNA may be biased, given the limitations of relying on a single genetic marker. Further studies should be oriented toward the use of SNP markers, enabling differentiation levels at putative candidate loci under selection (Cure et al. 2017). This is particularly important as fisheries or climate change may have caused changes in the frequency of selectively important traits that cannot be easily measured with the present genetic markers.

### 4.2 Gene Flow and Structure of *Nephrops*

Nuclear microsatellite results were largely consistent with the scenario stated above, as relevant barriers to dispersal within the inshore samples of the northern and central eastern Adriatic (18VE, 19VE and 19BR) were found by both spatial analyses (Structure and DAPC). These analyses indicated that samples from the Velebit and Brač channels were genetically differentiated from the middle and southern offshore localities, suggesting high larval retention and confined dispersal. Panmixia was observed in all offshore samples, whereas the northernmost sample from Kvarner Bay (19MD) showed a transition pattern from nearshore to offshore origin.

Still, considering the results of the Structure analysis at *K* = 2, which show no significant structuring among samples from the middle Adriatic, it can be concluded that there is some degree of gene flow between individuals from the Brač Channel and the neighbouring open seas. This gene flow is likely facilitated by the absence of clear physical or coastal oceanographic barriers in the middle Adriatic, in contrast to the more restricted gene flow observed among individuals from the Velebit Channel, with the Kvarner area acting as a mediator.

A similar connectivity pattern has been identified for the Kvarner area, as indicated by a Lagrangian model incorporating a specialised larval behaviour module along with environmental, hydrodynamic and meteorological data (Melaku Canu et al. 2021). This analysis suggests that, under specific oceanographic conditions, the Kvarner area exhibits spillover potential but receives successful settlers from a limited number of spawning sites. Melaku Canu et al. (2021) suggested the presence of at least three distinct subpopulations, referring to the populations from the northern Croatian coast as self‐sustained and highlighting a negligible connection between the Jabuka pit area and the area off Ancona, which reinforces the conclusion of a morphological study (Angelini et al. 2020). Due to different densities and growth rates between the subpopulations of Ancona and Jabuka Pit (Angelini et al. 2020; Carpi, Scarcella, and Cardinale 2017; Morello, Froglia, and Atkinson 2007), arguments have been made in the assessment and management of *Nephrops* populations in those areas. In the present study, no gene break in connectivity of the Ancona, Jabuka Pit or South Adriatic Pit subpopulations was noticed, suggesting the impact of environmental conditions as the primary driver of the observed phenotypic diversity.

Considering that few individuals per generation can maintain genetic homogeneity among populations (Woodruff 2001), additional sampling is needed, including temporal replicates along the western Adriatic coast, to obtain a representative pattern of genetic structure across the basin. This is especially true for species with long pelagic larval duration (3–7 weeks for *N. norvegicus*, see Melaku Canu et al. 2021), that is, species with high dispersal potential (Macpherson and Raventós 2006).

High potential spillover but limited dispersal indexes were attributed to the population from the Brač channel (Melaku Canu et al. 2021), supporting the findings of the present study that separated the Brač channel individuals into a separate genetic cluster at *K* = 3. This is additionally driven by the fact that both samples from the Velebit and Brač channels suffer from reduced contemporary *N*_e_ in contrast to open‐sea samples, limiting spillover potential. Contemporary and historical gene flow estimations demonstrated limited exchange of genetic material between inshore‐offshore areas, supporting the findings of a self‐sustained pattern of coastal Adriatic populations (Melaku Canu et al. 2021), where the significantly larger mutation‐scaled population size observed in offshore populations reflects more stable historical biogeographical conditions, characteristic of deeper waters.

At the contemporary level, it seems that 7 years of strong fisheries restrictions in the Jabuka Pit area in the central Adriatic, an important spawning and recruitment site for *Nephrops* (Melaku Canu et al. 2021; Chiarini et al. 2022), were beneficial in terms of genetic diversity and overall fitness maintenance of open‐sea populations. In contrast, the estimated overall effective population size of coastal individuals is at the limit of the minimum size for a viable population required to avoid population extinction in the long term (Pérez‐Pereira et al. 2022). Confirmed demographic changes where the majority of populations showed coherent signals of genetic erosion and experienced bottlenecks, stress the need for better management of coastal *Nephrops* populations.

### 4.3 Management Implications

One of the most effective strategies for preserving fisheries resources and marine biodiversity is the establishment of marine protected areas—designated geographic areas managed for the long‐term conservation of marine life. However, given the broader ecological, social, economic and political context of coastal and offshore areas, protecting only a small fraction of the sea can trigger significant positive effects in the surrounding areas.

Our study confirmed that the implementation of a fisheries restricted area in the Jabuka Pit in 2017—an area critical for the spawning and recruitment of *Nephrops* in the Adriatic Sea—yielded beneficial effects on genetic diversity and overall population fitness within a relatively short period. These findings align with previous research by Martinelli et al. (2023), highlighting the positive impact of management measures on epibenthic communities.

The distinct genetic structuring between northern inshore and offshore samples of *Nephrops norvegicus* observed in the Adriatic Sea underscores the importance of considering spatial population dynamics in fisheries management. Our findings suggest limited gene flow between these areas, potentially increasing *Nephrops* vulnerability to localised overfishing. While these results alone may not directly influence fisheries regulations, they provide a foundation for further research and potential future management actions.

Offshore *Nephrops* populations are harvested only by bottom trawlers, whereas coastal populations are targeted by both bottom trawlers and small‐scale fishers using creels (Morello et al. 2009; Brčić et al. 2018). Both fisheries are subjected to various spatial, temporal and technical regulations (Croatian Regulation 2017), with and a minimum conservation reference size mandated for *Nephrops* (Council Regulation (EC) No 1967/2006). In certain EU countries, offshore bottom trawling is banned (Ungfors et al. 2013), leaving coastal fishing grounds to more selective and less ecologically damaging techniques, such as creels (Eno et al. 2001), which are often cited as preferable alternatives to bottom trawls (Petetta et al. 2021).

The use of creel netting with a larger mesh size to improved selectivity (Mašanović, Herrmann, and Brčić 2022), combined with mandatory release of berried females (Merder et al. 2020) could enhance recruitment and increase effective population size. Such practice is mandated for the Scottish *Nephrops* fishery (Ungfors et al. 2013) and for several other commercially significant species, such as crawfish (*Palinurus* spp.) and European lobster (*H. gammarus*) in the Mediterranean Sea (Regulation (EU) 2019/1241).

As self‐sustained coastal populations are more prone to depletion due to the genetic diversity loss, we recommend incorporating genetic monitoring into the current management framework to provide ongoing assessments of population structure and diversity indices. Regular sampling and use of high‐resolution molecular marker, such as single‐nucleotide polymorphism (SNP), would be more effective in detecting changes over time. This approach would allow for the identification of early signs of stock depletion or shifts in population dynamics, thereby enabling timely management interventions.

## Author Contributions

**Marina Mašanović:** conceptualization (equal), data curation (lead), funding acquisition (lead), writing – review and editing (equal). **Luka Žuvić:** formal analysis (equal), investigation (equal), methodology (equal). **Iva Žužul:** formal analysis (equal), investigation (equal). **Igor Talijančić:** data curation (equal), methodology (equal). **Tanja Šegvić‐Bubić:** conceptualization (lead), investigation (equal), methodology (equal), software (lead), visualization (lead), writing – original draft (lead), writing – review and editing (equal).

## Conflicts of Interest

The authors declare no conflicts of interest.

## Supporting information


Data S1.

## Data Availability

Mitochondrial gene sequences in this study were deposited in NCBI's GenBank with the accession numbers PP478077‐PP478092. Microsatellite data supporting the findings of this study can be found in the Dryad data repository at https://datadryad.org/stash/share/2UIdDOFdjE‐4LFRLLaApZpIl2F5vyt2oKD6OkhtCwTo.

## References

[ece370358-bib-0001] Aguzzi, J. , R. Allué , and F. Sardà . 2004. “Characterization of Seasonal and Diel Variations in *Nephrops norvegicus* (Decapoda: Nephropidae) Landings off the Catalan Coasts.” Fishery Research 69: 293–300.

[ece370358-bib-0002] Aguzzi, J. , J. B. Company , and F. Sardà . 2007. “The Activity Rhythm of Berried and Unberried Females of *Nephrops norvegicus* (Crustacea, Decapoda).” Crustaceana 80, no. 9: 1121–1134.

[ece370358-bib-0003] Aguzzi, J. , and F. Sardà . 2008. “A History of Recent Advancements on *Nephrops norvegicus* Behavioral and Physiological Rhythms.” Reviews in Fish Biology and Fisheries 18: 235–248.

[ece370358-bib-0004] André, C. , and H. Knutsen . 2010. “Development of Twelve Novel Microsatellite Loci in the European Lobster (*Homarus gammarus*).” Conservation Genetics Resources 2, no. Suppl 1: 233–236. 10.1007/s12686-009-9151-3.

[ece370358-bib-0005] Angelini, S. , M. Martinelli , A. Santojanni , and S. Colella . 2020. “Biological Evidence of the Presence of Different Subpopulations of Norway Lobster (*Nephrops norvegicus*) in the Adriatic Sea (Central Mediterranean Sea).” Fisheries Research 221: 105365.

[ece370358-bib-0006] Assis, J. , N. C. Coelho , T. Lamy , M. Valero , F. Alberto , and E. Á. Serrão . 2016. “Deep Reefs Are Climatic Refugia for Genetic Diversity of Marine Forests.” Journal of Biogeography 43, no. 4: 833–844.

[ece370358-bib-0007] Beerli, P. 2006. “Comparison of Bayesian and Maximum‐Likelihood Inference of Population Genetic Parameters.” Bioinformatics 22: 341–345. 10.1093/bioinformatics/bti803. 16317072

[ece370358-bib-0009] Beerli, P. , and J. Felsenstein . 1999. “Maximum‐Likelihood Estimation of Migration Rates and Effective Population Numbers in Two Populations Using a Coalescent Approach.” Genetics 152: 763–773. 10353916 10.1093/genetics/152.2.763 PMC1460627

[ece370358-bib-0010] Beerli, P. , and M. Palczewski . 2010. “Unified Framework to Evaluate Panmixia and Migration Direction Among Multiple Sampling Locations.” Genetics 185, no. 1: 313–326. 20176979 10.1534/genetics.109.112532 PMC2870966

[ece370358-bib-0011] Benac, Č. , and M. Juračić . 1998. “Geomorphological Indicators of Sea Level Changes During Upper Pleistocene (Würm) and Holocene in the Kvarner Region (NE Adriatic Sea).” Acta Geographica Croatica 33, no. 1: 27–42.

[ece370358-bib-0013] Bianchi, C. N. , C. Morri , M. Chiantore , M. Montefalcone , V. Parravicini , and A. Rovere . 2012. “Mediterranean Sea Biodiversity Between the Legacy From the Past and a Future of Change.” In Life in the Mediterranean Sea: A Look at Habitat Changes, edited by N. Stambler , 1–55. New York, NY: Nova Science Publishers.

[ece370358-bib-0014] Bouckaert, R. , T. G. Vaughan , J. Barido‐Sottani , et al. 2019. “BEAST 2.5: An Advanced Software Platform for Bayesian Evolutionary Analysis.” PLoS Computational Biology 15: e1006650. 10.1371/journal.pcbi.1006650. 30958812 PMC6472827

[ece370358-bib-0015] Brčić, J. , B. Herrmann , M. Mašanović , M. Baranović , S. K. Šifner , and F. Škeljo . 2018. “Size Selection of *Nephrops norvegicus* (L.) in Commercial Creel Fishery in the Mediterranean Sea.” Fisheries Research 200: 25–32.

[ece370358-bib-0016] Carpi, P. , G. Scarcella , and M. Cardinale . 2017. “The Saga of the Management of Fisheries in the Adriatic Sea: History, Flaws, Difficulties, and Successes Toward the Application of the Common Fisheries Policy in the Mediterranean.” Frontiers in Marine Science 4: 423.

[ece370358-bib-0017] Chapuis, M. P. , and A. Estoup . 2007. “Microsatellite Null Alleles and Estimation of Population Differentiation.” Molecular Biology and Evolution 24: 621–631. 10.1093/molbev/msl191. 17150975

[ece370358-bib-0018] Chiarini, M. , S. Guicciardi , S. Angelini , I. D. Tuck , F. Grilli , and P. Penna . 2022. “Accounting for Environmental and Fishery Management Factors When Standardizing CPUE Data From a Scientific Survey: A Case Study for *Nephrops norvegicus* in the Pomo Pits Area (Central Adriatic Sea).” PLoS One 17, no. 7: e0270703. 10.1371/journal.pone.0270703. 35834483 PMC9282463

[ece370358-bib-0019] Cordero, D. , J. B. Peña , and C. Saavedra . 2014. “Phylogeographic Analysis of Introns and Mitochondrial DNA in the Clam *Ruditapes decussatus* Uncovers the Effects of Pleistocene Glaciations and Endogenous Barriers to Gene Flow.” Molecular Phylogenetics and Evolution 71: 274–287. 24269315 10.1016/j.ympev.2013.11.003

[ece370358-bib-0020] Council Regulation (EC) . “No 1967/2006 of 21 December 2006 Concerning Management Measures for the Sustainable Exploitation of Fishery Resources in the Mediterranean Sea, Amending Regulation (EEC) No 2847/93 and Repealing Regulation (EC) No 1626/94.”

[ece370358-bib-0021] Croatian Regulation . 2017. “Zakon o morskom ribarstvu NN 062/2017.” http://digarhiv.gov.hr/arhiva/263/168994/narodnenovine.nn.hr/clanci/sluzbeni/full/2017_06_62_1429.html.

[ece370358-bib-0022] Cure, K. , L. Thomas , J. P. A. Hobbs , D. V. Fairclough , and W. J. Kennington . 2017. “Genomic Signatures of Local Adaptation Reveal Source‐Sink Dynamics in a High Gene Flow Fish Species.” Scientific Reports 7: 8618. 10.1038/s41598-017-09224-y. 28819230 PMC5561064

[ece370358-bib-0023] Dickey‐Collas, M. , N. McQuaid , M. J. Armstrong , M. Allen , and R. P. Briggs . 2000. “Temperature‐Dependent Stage Durations of Irish Sea *Nephrops* Larvae.” Journal of Plankton Research 22, no. 4: 749–760.

[ece370358-bib-0024] Do, C. , R. S. Waples , D. Peel , G. M. Macbeth , B. J. Tillett , and J. R. Ovenden . 2014. “NeEstimator v2: Re‐Implementation of Software for the Estimation of Contemporary Effective Population Size (*Ne*) From Genetic Data.” Molecular Ecology Resources 14: 209–214. 10.1111/1755-0998.12157. 23992227

[ece370358-bib-0025] Drummond, A. J. , A. Rambaut , B. Shapiro , and O. G. Pybus . 2005. “Bayesian Coalescent Inference of Past Population Dynamics From Molecular Sequences.” Molecular Biology and Evolution 22: 1185–1192. 10.1093/molbev/msi103. 15703244

[ece370358-bib-0026] Earl, D. A. , and B. M. von Holdt . 2012. “STRUCTUREHARVESTER: A Website and Program for Visualizing STRUCTURE Output and Implementing the Evanno Method.” Conservation Genetics Resources 4: 359–361. 10.1007/s12686-011-9548-7.

[ece370358-bib-0027] Emig, C. C. , and P. Geistdoerfer . 2004. “The Mediterranean Deep‐Sea Fauna: Historical Evolution, Bathymetric Variations and Geographical Changes.” Carnets Geologiques 4, no. A01: 10. 10.4267/2042/3230.

[ece370358-bib-0028] Eno, N. C. , D. S. MacDonald , J. A. M. Kinnear , et al. 2001. “Effects of Crustacean Traps on Benthic Fauna.” ICES Journal of Marine Science 58: 11–20. 10.1006/jmsc.2000.0984.

[ece370358-bib-0205] European Commission, Joint Research Centre, Scientific, Technical and Economic Committee for Fisheries (STECF) . 2023. Mediterranean fisheries (STECF‐23‐15). Luxembourg: Publications Office of the European Union. 10.2760/192738.

[ece370358-bib-0030] Excoffier, L. , and H. E. Lischer . 2010. “Arlequin Suite ver 3.5: A New Series of Programs to Perform Population Genetics Analyses Under Linux and Windows.” Molecular Ecology Resources 10: 564–567. 21565059 10.1111/j.1755-0998.2010.02847.x

[ece370358-bib-0031] FAO . 2016. “General Fisheries Commission for the Mediterranean.” Report of the Eighteenth Session of the Scientific Advisory Committee on Fisheries; FAO Fisheries and Aquaculture Report No. 1154, Nicosia; Rome.

[ece370358-bib-0032] FAO . 2020. The State of Mediterranean and Black Sea Fisheries 2020. Rome: General Fisheries Commission for the Mediterranean.

[ece370358-bib-0034] FAO . 2023. “*Nephrops norvegicus* Linnaeus, 1758.” Fisheries and Aquaculture Division. https://www.fao.org/fishery/en/aqspecies/2647/en.

[ece370358-bib-0036] Farmer, A. S. D. 1974. “Burrowing Behaviour of the Norway Lobster, *Nephrops norvegicus* (L.) (Decapoda: Nephropidae).” Estuarine and Coastal Marine Science 2: 49–58.

[ece370358-bib-0037] Farmer, A. S. D. 1975. “Synopsis of Biological Data on the Norway Lobster *Nephrops norvegicus* (Linnaeus, 1758).” FAO Fisheries Synopsis 112: 1–97.

[ece370358-bib-0038] Froglia, C. , and M. E. Gramitto . 1981. “Summary of Biological Parameters on the Norway Lobster, *Nephrops norvegicus* (L.), in the Adriatic.” FAO Fisheries Report 253: 165–178.

[ece370358-bib-0039] Froglia, C. , and M. E. Gramitto . 1988. “An Estimate of Growth and Mortality Parameters for Norway Lobster (*Nephrops norvegicus*) in the Central Adriatic Sea.” FAO Fisheries Report 394: 189–203.

[ece370358-bib-0040] Fu, Y.‐X. 1997. “Statistical Tests of Neutrality of Mutations Against Population Growth, Hitchhiking and Background Selection.” Genetics 147: 915–925. 9335623 10.1093/genetics/147.2.915 PMC1208208

[ece370358-bib-0041] Gallagher, J. , J. Finarelli , J. Jonasson , and J. Carlsson . 2019. “Mitochondrial D‐Loop DNA Analyses of Norway Lobster (*Nephrops norvegicus*) Reveals Genetic Isolation Between Atlantic and East Mediterranean Populations.” Journal of the Marine Biological Association of the United Kingdom 99, no. 4: 933–940. 10.1017/S0025315418000929.

[ece370358-bib-0043] Goudet, J. 2022. “FSTAT, A Program to Estimate and Test Gene Diversities and Fixation Indices (Version 2.9.3.2).” https://www2.unil.ch/popgen/softwares/fstat.htm.

[ece370358-bib-0044] Haynes, P. S. , P. Browne , L. Fullbrook , et al. 2016. “Growth in *Nephrops norvegicus* From a Tag‐Recapture Experiment.” Scientific Reports 6: 35143. 27725735 10.1038/srep35143 PMC5057119

[ece370358-bib-0045] Huson, D. H. , and D. Bryant . 2006. “Application of Phylogenetic Networks in Evolutionary Studies.” Molecular Biology and Evolution 23, no. 2: 254–267. 16221896 10.1093/molbev/msj030

[ece370358-bib-0201] Johnson, M. P. , C. Lordan , and A. M. Power . 2013. “Habitat and Ecology of *Nephrops norvegicus* .” Advances in Marine Biology 64: 27–63. 10.1016/b978-0-12-410466-2.00002-9. 23668587

[ece370358-bib-0046] Jakobsson, M. , and N. A. Rosenberg . 2007. “CLUMPP: A Cluster Matching and Permutation Program for Dealing With Label Switching and Multimodality in Analysis of Population Structure.” Bioinformatics 23: 1801–1806. 10.1093/bioinformatics/btm233. 17485429

[ece370358-bib-0047] Jombart, T. 2008. “Adegenet: A R Package for the Multivariate Analysis of Genetic Markers.” Bioinformatics 24: 1403–1405. 10.1093/bioinformatics/btn129. 18397895

[ece370358-bib-0048] Kumar, S. , G. Stecher , K. Tamura , and J. Dudley . 2016. “MEGA7: Molecular Evolutionary Genetics Analysis Version 7.0 for Bigger Datasets.” Molecular Biology and Evolution 33: 1870–1874. 10.1093/molbev/msw054. 27004904 PMC8210823

[ece370358-bib-0049] Leigh, J. W. , and D. Bryant . 2015. “PopART: Full‐Feature Software for Haplotype Network Construction.” Methods in Ecology and Evolution 6, no. 9: 1110–1116.

[ece370358-bib-0050] Librado, P. , and J. Rozas . 2009. “DnaSP v5: A Software for Comprehensive Analysis of DNA Polymorphism Data.” Bioinformatics 25: 1451–1452. 10.1093/bioinformatics/btp187. 19346325

[ece370358-bib-0051] Macedo, D. , I. Caballero , M. Mateos , R. Leblois , S. McCay , and L. A. Hurtado . 2019. “Population Genetics and Historical Demographic Inferences of the Blue Crab *Callinectes sapidus* in the US Based on Microsatellites.” PeerJ 7: e7780. 31632846 10.7717/peerj.7780 PMC6796965

[ece370358-bib-0052] Macpherson, E. , and N. Raventós . 2006. “Relationship Between Pelagic Larval Duration and Geographic Distribution of Mediterranean Littoral Fishes.” Marine Ecology Progress Series 327: 257–265.

[ece370358-bib-0053] Maltagliati, F. , L. Camilli , F. Biagi , and M. Abbiati . 1998. “Genetic Structure of Norway Lobster, *Nephrops norvegicus* (L.) (Crustacea: Nephropidae), from the Mediterranean Sea.” Scientia Marina 62, no. S1: 91–99.

[ece370358-bib-0054] Maltagliati, F. , G. Di Giuseppe , M. Barbieri , A. Castelli , and F. Dini . 2010. “Phylogeography and Genetic Structure of the Edible Sea Urchin *Paracentrotus lividus* (Echinodermata: Echinoidea) Inferred From the Mitochondrial Cytochrome b Gene.” Biological Journal of the Linnean Society 100, no. 4: 910–923.

[ece370358-bib-0055] Mantovani, B. , and V. Scali . 1992. “Allozyme Characterization of the Norway Lobster, *Nephrops norvegicus*, of Two Adriatic Trawling Grounds.” Acta Adriatica 33, no. 1/2: 209–213.

[ece370358-bib-0056] Martinelli, M. , L. Zacchetti , A. Belardinelli , et al. 2023. “Changes in Abundance and Distribution of the Sea Pen, *Funiculina quadrangularis*, in the Central Adriatic Sea (Mediterranean Basin) in Response to Variations in Trawling Intensity.” Fishes 8: 347. 10.3390/fishes8070347.

[ece370358-bib-0057] Mašanović, M. , B. Herrmann , and J. Brčić . 2022. “Escape, Discard, and Landing Probability of *Nephrops norvegicus* in the Mediterranean Sea Creel Fishery.” Canadian Journal of Fisheries and Aquatic Sciences 80, no. 4: 719–731.

[ece370358-bib-0058] Melaku Canu, D. , C. Laurent , E. B. Morello , et al. 2021. “ *Nephrops norvegicus* in the Adriatic Sea: Connectivity Modeling, Essential Fish Habitats, and Management Area Network.” Fisheries Oceanography 30: 349–365. 10.1111/fog.12522.

[ece370358-bib-0059] Merder, J. , P. Browne , J. A. Freund , et al. 2020. “Density‐Dependent Growth in ‘Catch‐And‐Wait’ Fisheries Has Implications for Fisheries Management and Marine Protected Areas.” Ambio 49: 107–117. 30852778 10.1007/s13280-019-01158-1 PMC6889112

[ece370358-bib-0060] Miller, M. A. , W. Pfeiffer , and T. Schwartz . 2010. “Creating the CIPRES Science Gateway for Phylogenetic Research.” In *Proceedings of the 2010 Gateway Computing Environments Workshop* (*GCE*), 1–8.

[ece370358-bib-0061] Morello, E. B. , B. Antolini , M. E. Gramitto , R. J. A. Atkinson , and C. Froglia . 2009. “The Fishery for *Nephrops norvegicus* (Linnaeus, 1758) in the Central Adriatic Sea (Italy): Preliminary Observations Comparing Bottom Trawl and Baited Creels.” Fisheries Research 95: 325–331.

[ece370358-bib-0062] Morello, E. B. , C. Froglia , and R. J. A. Atkinson . 2007. “Underwater Television as a Fishery‐Independent Method for Stock Assessment of Norway Lobster (*Nephrops norvegicus*) in the Central Adriatic Sea (Italy).” ICES Journal of Marine Science 64, no. 6: 1116–1123.

[ece370358-bib-0063] Mytilineou, C. , M. Castro , P. Gancho , and A. Fourtouni . 1998. “Growth Studies on Norway Lobster, *Nephrops norvegicus* (L.), in Different Areas of the Mediterranean Sea and the Adjacent Atlantic.” Scientia Marina 62: 43–60.

[ece370358-bib-0064] Naylor, E. 2010. Chronobiology of Marine Organisms. Cambridge: Cambridge University Press.

[ece370358-bib-0065] Novosel, M. , T. Bakran‐Petricioli , A. Požar‐Domac , P. Kružić , and I. Radić . 2002. “The Benthos of the Northern Part of the Velebit Channel (Adriatic Sea, Croatia).” Natura Croatica: Periodicum Musei Historiae Naturalis Croatici 11, no. 4: 387–409.

[ece370358-bib-0066] Orlić, M. , M. Gačić , and P. E. LaViolette . 2012. “The Currents and Circulation of the Adriatic Sea.” Oceanologica Acta 15: 109–124.

[ece370358-bib-0067] Pampoulie, C. , S. Skirnisdottir , S. Hauksdottir , et al. 2011. “A Pilot Genetic Study Reveals the Absence of Spatial Genetic Structure in Norway Lobster (*Nephrops norvegicus*) on Fishing Grounds in Icelandic Waters.” ICES Journal of Marine Science 68, no. 1: 20–25.

[ece370358-bib-0068] Patarnello, T. , F. A. M. J. Volckaert , and R. Castilho . 2007. “Pillars of Hercules: Is the Atlantic–Mediterranean Transition a Phylogeographical Break?” Molecular Ecology 16: 4426–4444. 17908222 10.1111/j.1365-294X.2007.03477.x

[ece370358-bib-0069] Pavičić, M. , I. Žužul , S. Matić‐Skoko , et al. 2020. “Population Genetic Structure and Connectivity of the European Lobster *Homarus gammarus* in the Adriatic and Mediterranean Seas.” Frontiers in Genetics 11: 1540. 10.3389/fgene.2020.576023 PMC7750201 33365046

[ece370358-bib-0070] Pérez‐Losada, M. , M. J. Nolte , K. A. Crandall , and P. W. Shaw . 2007. “Testing Hypotheses of Population Structuring in the Northeast Atlantic Ocean and Mediterranean Sea Using the Common Cuttlefish *Sepia officinalis* .” Molecular Ecology 16, no. 13: 2667–2679. 10.1111/j.1365-294X.2007.03333.x. 17594438

[ece370358-bib-0071] Pérez‐Pereira, N. , J. Wang , H. Quesada , and A. Caballero . 2022. “Prediction of the Minimum Effective Size of a Population Viable in the Long Term.” Biodiversity and Conservation 31: 2763–2780. 10.1007/s10531-022-02456-z.

[ece370358-bib-0150] Petetta, A. , M. Virgili , S. Guicciardi , and A. Lucchetti . 2021. “Pots as Alternative and Sustainable Fishing Gears in the Mediterranean Sea: An Overview.” Reviews in Fish Biology and Fisheries 31: 773–795. 10.1007/s11160-021-09676-6.

[ece370358-bib-0073] Piry, S. , G. Luikart , and J.‐M. Cornuet . 1999. “BOTTLENECK: A Computer Program for Detecting Recent Reductions in the Effective Size Using Allele Frequency Data.” Journal of Heredity 90: 502–503. 10.1093/jhered/90.4.502.

[ece370358-bib-0074] Piva, A. , A. Asioli , F. Trincardi , R. R. Schneider , and L. Vigliotti . 2008. “Late‐Holocene Climate Variability in the Adriatic Sea (Central Mediterranean).” Holocene 18, no. 1: 153–167. 10.1177/0959683607085606.

[ece370358-bib-0075] Powell, A. , and S. P. Eriksson . 2013. “Reproduction: Life Cycle, Larvae and Larviculture.” Advances in Marine Biology 64: 201–245. 10.1016/B978-0-12-410466-2.00006-6. 23668591

[ece370358-bib-0076] Pritchard, J. K. , M. Stephens , and P. Donnelly . 2000. “Inference of Population Structure Using Multilocus Genotype Data.” Genetics 155: 945–959. 10835412 10.1093/genetics/155.2.945 PMC1461096

[ece370358-bib-0077] R Core Team . 2017. R: A Language and Environment for Statistical Computing. Vienna: R Foundation for Statistical Computing.

[ece370358-bib-0078] Rambaut, A. , A. J. Drummond , D. Xie , G. Baele , and M. A. Suchard . 2018. “Posterior Summarization in Bayesian Phylogenetics Using Tracer 1.7.” Systematic Biology 67: 901–904. 10.1093/sysbio/syy032. 29718447 PMC6101584

[ece370358-bib-0079] Regulation (EU) . “2019/1241 of the European Parliament and of the Council of 20 June 2019 on the Conservation of Fisheries Resources and the Protection of Marine Ecosystems Through Technical Measures.” http://data.europa.eu/eli/reg/2019/1241/oj.

[ece370358-bib-0080] Relini, L. O. , A. Zamboni , F. Fiorentino , and D. Massi . 1998. “Reproductive Patterns in Norway Lobster (*Nephrops norvegicus* L., Crustacea Decapoda Nephropidae) of Different Mediterranean Areas.” Scientia Marina 62, no. S1: 25–41.

[ece370358-bib-0204] Rice, W. R. 1989. “Analyzing Tables of Statistical Tests.” Evolution 1: 223–225. 10.1111/j.1558-5646.1989.tb04220.x 28568501

[ece370358-bib-0081] Rosenberg, N. A. 2004. “DISTRUCT: A Program for the Graphical Display of Population Structure.” Molecular Ecology Notes 4: 137–138. 10.1046/j.1471-8286.2003.00566.x.

[ece370358-bib-0203] Rousset, F. 2008. “Genepop’007: A Complete Re‐Implementation of the Genepop Software for Windows and Linux.” Molecular Ecology Resources 8, no. 1: 103–106. 21585727 10.1111/j.1471-8286.2007.01931.x

[ece370358-bib-0082] Ryman, N. , and S. Palm . 2006. “POWSIM: A Computer Program for Assessing Statistical Power When Testing for Genetic Differentiation.” Molecular Ecology Notes 6: 600–602. 10.1111/j.1471-8286.2006.01378.x. 11703649

[ece370358-bib-0083] Sabatini, L. , M. Bullo , A. Cariani , et al. 2018. “Good Practices for Common Sole Assessment in the Adriatic Sea: Genetic and Morphological Differentiation of *Solea Solea* (Linnaeus, 1758) From *Solea aegyptiaca* and Stock Identification.” Journal of Sea Research 137: 57–64.

[ece370358-bib-0084] Skirnisdottir, S. , K. Olafsson , S. Hauksdottir , et al. 2010. “Isolation and Characterization of Eight New Microsatellite Loci in the Norway Lobster, *Nephrops norvegicus* (Linnaeus, 1758).” Molecular Ecology Resources Database. http://tomato.bio.trinity.edu/manuscripts/10‐4/mer‐10‐0048.pdf.

[ece370358-bib-0085] Spedicato, M. T. , R. Cannas , K. Mahé , et al. 2022. “Study on Advancing Fisheries Assessment and Management Advice in the Mediterranean by Aligning Biological and Management Units of Priority Species (MED_UNITs).” *Final Report*. European Commission‐ European Climate, Infrastructure and Environmental Executive Agency, Publications Office of the European Union, Luxembourg. 10.2926/909535.

[ece370358-bib-0086] Sponaugle, S. , R. K. Cowen , A. Shanks , et al. 2002. “Predicting Self‐Recruitment in Marine Populations: Biophysical Correlates and Mechanisms.” Bulletin of Marine Science 70, no. 1: 341–375.

[ece370358-bib-0087] Stamatis, C. , A. Triantafyllidis , K. A. Moutou , and Z. Mamuris . 2004. “Mitochondrial DNA Variation in Northeast Atlantic and Mediterranean Populations of Norway Lobster, *Nephrops norvegicus* .” Molecular Ecology 13, no. 6: 1377–1390. 15140084 10.1111/j.1365-294X.2004.02165.x

[ece370358-bib-0088] Stamatis, C. , A. Triantafyllidis , K. A. Moutou , and Z. Mamuris . 2006. “Allozymic Variation in Northeast Atlantic and Mediterranean Populations of Norway Lobster, *Nephrops norvegicus* .” ICES Journal of Marine Science 63, no. 5: 875–882. 10.1111/j.1365-294X.2004.02165.x 15140084

[ece370358-bib-0202] STECF . 2015. Scientific, Technical and Economic Committee for Fisheries (STECF) ‐ Mediterranean Assessments ‐ Part 2 (STECF‐15‐06). EUR 27221 EU. Luxembourg: Publications Office of the European Union. 10.2788/20044.

[ece370358-bib-0089] STECF . 2020. “Technical and Economic Committee for Fisheries, Scientific.” In Stock Assessments in the Mediterranean Sea ‐ Adriatic, Ionian and Aegean Seas (STECF‐20‐15). EUR 28359 EU, edited by J. Simmonds , C. Pinto , and A. Mannini . Luxembourg: Publications Office of the European Union. 10.2760/877405.

[ece370358-bib-0090] STECF . 2022. “European Commission, Joint Research Centre, Scientific, Technical and Economic Committee for Fisheries.” In The 2022 Annual Economic Report on the EU Fishing Fleet (STECF 22‐06), edited by J. Virtanen , J. Guillen , R. Prellezo , and E. Sabatella . Luxembourg: Publications Office of the European Union. 10.2760/120462.

[ece370358-bib-0092] Streiff, R. , T. Guillemaud , F. Alberto , J. Magalhaes , M. Castro , and M. L. Cancela . 2001. “Isolation and Characterization of Microsatellite Loci in the Norway Lobster (*Nephrops norvegicus*).” Molecular Ecology Notes 1: 71–72.

[ece370358-bib-0093] Streiff, R. , S. Mira , M. Castro , and M. L. Cancela . 2004. “Multiple Paternity in Norway Lobster (*Nephrops norvegicus* L.) Assessed With Microsatellite Markers.” Marine Biotechnology 6: 60–66. 14564537 10.1007/s10126-003-0015-7

[ece370358-bib-0094] Tajima, F. 1989. “Statistical Method for Testing the Neutral Mutation Hypothesis by DNA Polymorphism.” Genetics 123: 585–595. 2513255 10.1093/genetics/123.3.585 PMC1203831

[ece370358-bib-0095] Trincardi, A. , M. Correggiari , and M. Roveri . 1996. “Late Pleistocene and Holocene Evolution of the North Adriatic Sea.” Il Quaternario 9, no. 2: 697–704.

[ece370358-bib-0096] Ungfors, A. , E. Bell , M. L. Johnson , et al. 2013. “Nephrops Fisheries in European Waters.” Advances in Marine Biology 64: 247–314. 10.1016/B978-0-12-410466-2.00007-8. 23668592

[ece370358-bib-0097] Van Oosterhout, C. , W. F. Hutchinson , D. P. M. Wills , and P. Shipley . 2004. “MICRO‐CHECKER: Software for Identifying and Correcting Genotyping Errors in Microsatellite Data.” Molecular Ecology Notes 4: 535–538. 10.1111/j.1471-8286.2004.00684.x.

[ece370358-bib-0098] Vrgoč, N. , E. Arneri , S. Jukić‐Peladić , et al. 2004. “Review of Current Knowledge on Shared Demersal Stocks of the Adriatic Sea.” AdriaMed Technical Documents 12: 67–74.

[ece370358-bib-0099] Waples, R. S. , and C. Do . 2008. “LDNE: A Program for Estimating Effective Population Size From Data on Linkage Disequilibrium.” Molecular Ecology Resources 8: 753–756. 21585883 10.1111/j.1755-0998.2007.02061.x

[ece370358-bib-0100] Westgaard, J. I. , G. Søvik , and T. Johansen . 2023. “Genetic Population Structure in Norway Lobster (*Nephrops norvegicus*): Management Regime Under Panmixia.” ICES Journal of Marine Science 80, no. 4: 766–774.

[ece370358-bib-0101] Whittaker, J. C. , R. M. Harbord , N. Boxall , I. Mackay , G. Dawson , and R. M. Sibly . 2003. “Likelihood‐Based Estimation of Microsatellite Mutation Rates.” Genetics 164, no. 2: 781–787. 12807796 10.1093/genetics/164.2.781 PMC1462577

[ece370358-bib-0102] Wilson, G. A. , and B. Rannala . 2003. “Bayesian Inference of Recent Migration Rates Using Multilocus Genotypes.” Genetics 163: 1177–1191. 12663554 10.1093/genetics/163.3.1177 PMC1462502

[ece370358-bib-0162] Woodruff, D. S. 2001. “Populations, Species, and Conservation Genetics.” Encyclopedia of Biodiversity 811–829. 10.1016/B0-12-226865-2/00355-2.

[ece370358-bib-0104] Yeh, F. C. Y. C. , R. Yang , and T. R. Boyle . 1999. POPGENE Version 1.32 Microsoft Windows‐Based Freeware for Populations Genetic Analysis. Edmonton, AB: University of Alberta.

