# Supplementary material for: Seascape Genetics and Distinct Intraspecific Diversification of the Decapod Nephrops norvegicus in the Adriatic Sea
Source: Ecol Evol. 2024 Oct 8;14(10):e70358. doi: 10.1002/ece3.70358 (PMC11459092; doi:10.1002/ece3.70358)
Supplement: Supplementary file 1 — Data S1. [file ECE3-14-e70358-s001.docx]

**Seascape genetics and distinct intraspecific diversification of the decapod *Nephrops norvegicus* in the Adriatic Sea**

**Appendix S1**

**Supplementary methods: estimates of divergence dates using BEAST**

The data set contained 16 haplotypes of *Nephros norvegicus* and haplotypes of *Homarus americanus* and *Homarus gammarus*. D-loop sequences from other lobster taxa were not available in Genebank. Therefore, a calibration point at the tree root for the most recent common ancestor of a group of taxa was used to estimate divergence data. Because fossil records from the Mediterranean Sea were not available for the lobster record, we used calibration based on the molecular date estimates of Bracken-Grissom et al. (2014). A normal prior (mean 17 Ma, standard deviation 1.5) was set for the root of the tree, representing the divergence between sister taxa *Homarus* sp. and *Nephrops* sp. Because the present study is looking for events within species coalescence, a coalescent constant prior was applied to reflect that most haplotypes follow coalescent events. Molecular clock models were first evaluated using the ucld.stdev and coefficient of variation parameters, applying the uncorrelated relaxed lognormal clock prior and examining the resulting distribution (Drummond et al. 2007). Since the ucld.stdev and coefficient of variation parameters were close to 0, the data were considered to be clock-like. Therefore, a strict clock using the Tamura-Nei model (TrN+G) with four gamma categories was used. The optimal nucleotide substitution model was estimated using MODELTEST v3.7 (Posada and Crandall 1998) considering the AIC. Each run included 500 million Markov Chain Monte Carlo (MCMC) iterations sampled every 10,000 steps after an initial burn-in of 10%. The run was considered valid if all parameters had an effective population size > 200 in Tracer v1.7.1 (Rambaut et al., 2018). Trees were annotated with TreeAnnotator and visualised in Figtree v1.4.4 (Rambaut, 2009).

**Supplementary tables and figures**

**Table S1.1**. Primer sequences, GenBank accession number, dyes, number of multiplex reactions (Mix) and references for each microsatellite locus used for *Nephrops norvegicus* analysis are given. An annealing temperature of 57°C was set for all multiplex reactions.

| **Primer code** | **Acc. No.** | **Dye** | **Mix** | **Primer sequence (5’-3’)** | **Reference** |
| --- | --- | --- | --- | --- | --- |
| PLH33 | GU559888 | FAM | 1 | GATAATTCATTGTGCTAAGGTGG | Skirnisdottir et al., 2010 |
|  |  |  |  | TGTATACCTGTGTCTAACTGTG |  |
| Nnmic2-E4 | AF221987 | VIC | 1 | GGCGTGATGTCCGATTTTACTG | Streiff et al., 2001 |
|  |  |  |  | AGTAATGCCATCGCCGCTGTAGG |  |
| PLH46 | GU559890 | VIC | 1 | F: CCCTAGGATGGTGTCTTATCTC | Skirnisdottir et al., 2010 |
|  |  |  |  | R: GAGTCTTGTGTTATGGCAGC |  |
| PLH35 | GU559889 | NED | 1 | F: TAGTAATACCCAGGCGAGAA | Skirnisdottir et al., 2010 |
|  |  |  |  | R: GAGAAGGTGGACCGTAAAC |  |
| PLH15 | GU559886 | PET | 1 | F: CAATGGACGAAGATGAAATTGC | Skirnisdottir et al., 2010 |
|  |  |  |  | R: CACGTCGTGTTGACTCATTC |  |
| Nnmic1-C12 | AF221990 | FAM | 2 | TGTCGTCGTAACTACCCGCTG | Streiff et al., 2001 |
|  |  |  |  | ATACATCGTCCACTGCTCCAC |  |
| HGD106 |  | FAM | 2 | F: CATACCGAACCAAGTGTAAAC | André and Knutsen, 2010 |
|  |  |  |  | R: GCCCACAGTAACAGATAAGAG |  |
| PLH4 | GU559883 | VIC | 2 | F: TTGTACGGTACTTGTAGTGTAGG | Skirnisdottir et al., 2010 |
|  |  |  |  | R: ATGCTGATCCAATCATAAACAAG |  |
| Nnmic1B11 | AF221989 | PET | 2 | TACAACAAAGGGTCACTACAC | Streiff et al., 2001 |
|  |  |  |  | CCATAGAGCTAACTAACGCAGG |  |
| PLH5 | GU559884 | PET | 2 | F: CTATTACTCAGGATGCTTACGTG | Skirnisdottir et al., 2010 |
|  |  |  |  | R: CAAAGGGTTAACAGAGGTGTG |  |
| PLH31 | GU559887 | NED | 3 | F: ATTTCTAATTAAGGCCTCGACAG | Skirnisdottir et al., 2010 |
|  |  |  |  | R: TAACAGACAAGGTTCCTCTCC |  |
| NnmicT-G2 | AF221191 | NED | 3 | ACTGTCCGGTCAGGTTGAGA | Streiff et al., 2001 |
|  |  |  |  | TCAGTCAAGAGATTGGGGAG |  |

**Table S1.2.** Pairwise *F*_ST_ values based on D-loop haplotypes among eight samples of *Nephrops norvegicus* sampled in the Adriatic Sea. Significant *F*_ST_ values at *p* < 0.05 are shown in bold. Population codes are explained in Table 1.

|  | 19MD | 19VE | 19BR | 19AN | 19JA | 19PA | 19JK |
| --- | --- | --- | --- | --- | --- | --- | --- |
| 19VE | 0.01835 |  |  |  |  |  |  |
| 19BR | **0.13624** | **0.18348** |  |  |  |  |  |
| 19AN | **0.11195** | 0.07313 | -0.0218 |  |  |  |  |
| 19JA | **0.12993** | **0.21854** | -0.0526 | 0.05077 |  |  |  |
| 19PA | **0.33593** | **0.35410** | 0.09637 | 0.12591 | **0.19333** |  |  |
| 19JK | **0.15954** | **0.18662** | -0.0519 | -0.0164 | -0.0147 | 0.10833 |  |
| 19AL | **0.09338** | **0.11068** | -0.0083 | -0.0382 | 0.05888 | 0.05794 | -0.0049 |

**Table S1.3.** Summery statistics of 10 neutral microsatellite loci among samples of Norway lobster *Nephrops norvegicus.*

| Locus |  | 19MD | 18VE | 19VE | 18BR | 19BR | 19AN | 18JA | 19JA | 18PA | 19PA | 19JK | 19AL |
| --- | --- | --- | --- | --- | --- | --- | --- | --- | --- | --- | --- | --- | --- |
| Nnmic2-E4 | n | 30 | 35 | 60 | 24 | 42 | 46 | 35 | 30 | 46 | 55 | 41 | 38 |
|  | nA | 14 | 10 | 12 | 11 | 14 | 15 | 14 | 13 | 14 | 12 | 14 | 14 |
|  | HO | 0.966 | 0.829 | 0.833 | 0.916 | 0.929 | 0.826 | 0.771 | 0.966 | 0.978 | 0.818 | 0.829 | 0.763 |
|  | HE | 0.917 | 0.861 | 0.866 | 0.865 | 0.849 | 0.872 | 0.861 | 0.902 | 0.883 | 0.855 | 0.879 | 0.875 |
|  | FIS | -0.055 | 0.038 | 0.038 | -0.061 | -0.095 | 0.053 | 0.105 | -0.073 | -0.110 | 0.043 | 0.057 | 0.129 |
| PLH46 | n | 30 | 35 | 60 | 24 | 42 | 46 | 35 | 30 | 45 | 55 | 41 | 38 |
|  | nA | 24 | 20 | 28 | 21 | 20 | 27 | 22 | 19 | 22 | 29 | 22 | 22 |
|  | HO | 0.9333 | 0.914 | 0.933 | 1.00 | 0.929 | 0.913 | 0.914 | 0.933 | 0.911 | 0.964 | 0.902 | 0.974 |
|  | HE | 0.949 | 0.945 | 0.958 | 0.958 | 0.932 | 0.954 | 0.932 | 0.942 | 0.944 | 0.960 | 0.950 | 0.947 |
|  | FIS | 0.016 | 0.032 | 0.026 | -0.044 | 0.003 | 0.044 | 0.019 | 0.009 | 0.035 | -0.004 | 0.051 | -0.029 |
| PLH35 | n | 29 | 35 | 57 | 24 | 41 | 46 | 35 | 29 | 46 | 55 | 40 | 37 |
|  | nA | 22 | 22 | 27 | 26 | 31 | 37 | 32 | 26 | 33 | 33 | 34 | 32 |
|  | HO | 0.931 | 1.000 | 0.877 | 0.916 | 0.854 | 0.891 | 0.829 | 0.897 | 0.913 | 0.836 | 0.975 | 0.892 |
|  | HE | 0.948 | 0.949 | 0.948 | 0.965 | 0.968 | 0.969 | 0.959 | 0.961 | 0.967 | 0.966 | 0.973 | 0.967 |
|  | FIS | 0.018 | -0.054 | 0.076 | 0.051 | 0.120 | 0.081 | 0.137 | 0.068 | 0.057 | 0.136 | -0.002 | 0.079 |
| PLH15 | n | 30 | 35 | 58 | 24 | 39 | 44 | 34 | 30 | 46 | 55 | 40 | 37 |
|  | nA | 18 | 20 | 23 | 20 | 18 | 25 | 21 | 22 | 24 | 25 | 21 | 23 |
|  | HO | 0.766 | 0.971 | 0.776 | 1.000 | 0.795 | 0.841 | 0.853 | 0.766 | 0.957 | 0.800 | 0.875 | 0.757 |
|  | HE | 0.938 | 0.945 | 0.943 | 0.949 | 0.926 | 0.950 | 0.939 | 0.934 | 0.948 | 0.936 | 0.938 | 0.945 |
|  | FIS | 0.186 | -0.029 | 0.178 | 0.054 | 0.143 | 0.116 | 0.092 | 0.182 | -0.009 | 0.146 | 0.068 | 0.202 |
| HGD106 | n | 30 | 35 | 60 | 24 | 42 | 45 | 35 | 30 | 46 | 55 | 41 | 36 |
|  | nA | 8 | 7 | 10 | 9 | 8 | 9 | 9 | 7 | 10 | 8 | 9 | 7 |
|  | HO | 0.766 | 0.800 | 0.800 | 0.916 | 0.762 | 0.755 | 0.886 | 0.533 | 0.826 | 0.800 | 0.805 | 0.833 |
|  | HE | 0.765 | 0.749 | 0.789 | 0.833 | 0.769 | 0.817 | 0.803 | 0.807 | 0.840 | 0.815 | 0.783 | 0.822 |
|  | FIS | -0.002 | -0.068 | -0.013 | -0.102 | 0.011 | 0.076 | -0.105 | 0.343 | 0.017 | 0.018 | -0.028 | -0.013 |
| PLH4 | n | 30 | 35 | 60 | 24 | 42 | 46 | 35 | 30 | 46 | 55 | 41 | 38 |
|  | nA | 6 | 8 | 8 | 6 | 8 | 8 | 7 | 9 | 11 | 8 | 9 | 8 |
|  | HO | 0.666 | 0.857 | 0.683 | 0.625 | 0.786 | 0.695 | 0.686 | 0.633 | 0.652 | 0.836 | 0.829 | 0.658 |
|  | HE | 0.791 | 0.799 | 0.815 | 0.748 | 0.802 | 0.772 | 0.775 | 0.819 | 0.785 | 0.821 | 0.811 | 0.798 |
|  | FIS | 0.159 | -0.073 | 0.163 | 0.168 | 0.021 | 0.100 | 0.117 | 0.230 | 0.171 | -0.018 | -0.023 | 0.177 |
| Nnmic1B11 | n | 30 | 35 | 60 | 24 | 42 | 46 | 35 | 30 | 45 | 55 | 41 | 38 |
|  | nA | 9 | 12 | 17 | 13 | 11 | 15 | 11 | 13 | 18 | 19 | 13 | 16 |
|  | HO | 0.833 | 0.829 | 0.833 | 0.875 | 0.809 | 0.717 | 0.829 | 0.666 | 0.822 | 0.836 | 0.707 | 0.763 |
|  | HE | 0.814 | 0.834 | 0.858 | 0.853 | 0.795 | 0.864 | 0.819 | 0.808 | 0.8871 | 0.852 | 0.827 | 0.869 |
|  | FIS | -0.025 | 0.007 | 0.029 | -0.025 | -0.019 | 0.171 | -0.012 | 0.177 | 0.056 | 0.018 | 0.146 | 0.124 |
| PLH5 | n | 30 | 35 | 60 | 23 | 41 | 46 | 35 | 30 | 46 | 54 | 41 | 37 |
|  | nA | 10 | 11 | 11 | 8 | 9 | 12 | 10 | 8 | 11 | 12 | 12 | 11 |
|  | HO | 0.733 | 0.571 | 0.700 | 0.696 | 0.756 | 0.761 | 0.714 | 0.766 | 0.695 | 0.741 | 0.781 | 0.595 |
|  | HE | 0.848 | 0.677 | 0.804 | 0.805 | 0.812 | 0.832 | 0.762 | 0.848 | 0.813 | 0.829 | 0.842 | 0.793 |
|  | FIS | 0.137 | 0.158 | 0.130 | 0.069 | 0.069 | 0.086 | 0.064 | 0.096 | 0.146 | 0.108 | 0.074 | 0.252 |
| PLH31 | n | 24 | 35 | 58 | 24 | 41 | 44 | 34 | 29 | 45 | 52 | 40 | 35 |
|  | nA | 9 | 11 | 12 | 9 | 12 | 12 | 10 | 12 | 11 | 15 | 14 | 14 |
|  | HO | 0.788 | 0.629 | 0.586 | 0.708 | 0.829 | 0.500 | 0.824 | 0.759 | 0.488 | 0.692 | 0.825 | 0.829 |
|  | HE | 0.779 | 0.773 | 0.739 | 0.824 | 0.833 | 0.759 | 0.799 | 0.849 | 0.730 | 0.848 | 0.812 | 0.870 |
|  | FIS | 0.093 | 0.189 | 0.208 | 0.143 | 0.005 | 0.344 | -0.032 | 0.108 | 0.333 | 0.184 | -0.017 | 0.010 |
| NnmicT-G2 | n | 28 | 34 | 30 | 19 | 42 | 45 | 34 | 29 | 35 | 54 | 40 | 35 |
|  | nA | 25 | 23 | 29 | 18 | 29 | 27 | 30 | 26 | 26 | 31 | 28 | 29 |
|  | HO | 0.964 | 1.000 | 0.950 | 1.000 | 0.976 | 0.955 | 0.971 | 1.000 | 0.886 | 0.963 | 1.000 | 0.914 |
|  | HE | 0.966 | 0.961 | 0.958 | 0.953 | 0.962 | 0.962 | 0.973 | 0.963 | 0.959 | 0.968 | 0.964 | 0.968 |
|  | FIS | 0.002 | -0.042 | 0.009 | -0.051 | -0.015 | 0.007 | 0.003 | -0.039 | 0.077 | 0.005 | -0.038 | 0.056 |

**Table S1.4.** Pairwise *F*_ST_ values based on 10 microsatellites loci (below diagonal) among 12 samples of Norway lobster *Nephrops norvegicus* sampled in the Adriatic Sea. Significant *F*_ST_ values shown in bold at *p* < 0.0007 (Bonferroni correction). Population codes are explained in Table 1.

|  | **19MD** | **18VE** | **19VE** | **18HV** | **19BR** | **19AN** | **18JA** | **19JA** | **18PA** | **19PA** | **19JK** |
| --- | --- | --- | --- | --- | --- | --- | --- | --- | --- | --- | --- |
| **19MD** |  |  |  |  |  |  |  |  |  |  |  |
| **18VE** | 0.011 |  |  |  |  |  |  |  |  |  |  |
| **19VE** | 0.010 | **0.020** |  |  |  |  |  |  |  |  |  |
| **18BR** | 0.008 | 0.012 | **0.026** |  |  |  |  |  |  |  |  |
| **19BR** | **0.025** | **0.043** | **0.009** | **0.036** |  |  |  |  |  |  |  |
| **19AN** | 0.006 | **0.020** | **0.014** | 0.011 | **0.029** |  |  |  |  |  |  |
| **18JA** | 0.006 | **0.023** | **0.014** | 0.010 | **0.029** | 0.005 |  |  |  |  |  |
| **19JA** | 0.009 | **0.026** | **0.014** | 0.011 | **0.026** | **0.015** | **0.013** |  |  |  |  |
| **18PA** | **0.056** | **0.065** | **0.039** | **0.053** | **0.044** | **0.061** | **0.060** | **0.042** |  |  |  |
| **19PA** | 0.003 | **0.019** | **0.011** | 0.006 | **0.023** | 0.003 | 0.003 | 0.003 | **0.053** |  |  |
| **19JK** | 0.004 | **0.021** | **0.011** | **0.013** | **0.021** | 0.002 | 0.005 | **0.011** | **0.058** | 0.003 |  |
| **19AL** | 0.008 | **0.029** | 0.008 | **0.016** | **0.018** | 0.010 | 0.005 | 0.007 | **0.049** | 0.004 | 0.005 |

**Table S1.5.** Posterior distribution table of the winning model, with 95% CI for inshore (INS) and offshore (OFF) group parameters. ANC stands for introduced ancestral (‘ghost’) population.

| Locus | Parameter | 2.50% | 25.00% | Mode | 75.00% | 97.50% | Median | Mean |
| --- | --- | --- | --- | --- | --- | --- | --- | --- |
| All | Θ_INS_ | 1.200 | 3.000 | 4.100 | 5.133 | 6.933 | 4.166 | 4.112 |
| All | Θ_OFF_ | 1.800 | 4.333 | 5.500 | 6.666 | 11.200 | 5.833 | 7.398 |
| All | D_ANC_->INS | 0.000 | 0.280 | 0.780 | 1.240 | 2.120 | 1.020 | 0.775 |
| All | D_ANC_->OFF | 0.000 | 0.400 | 0.940 | 1.440 | 2.320 | 1.140 | 0.959 |

| 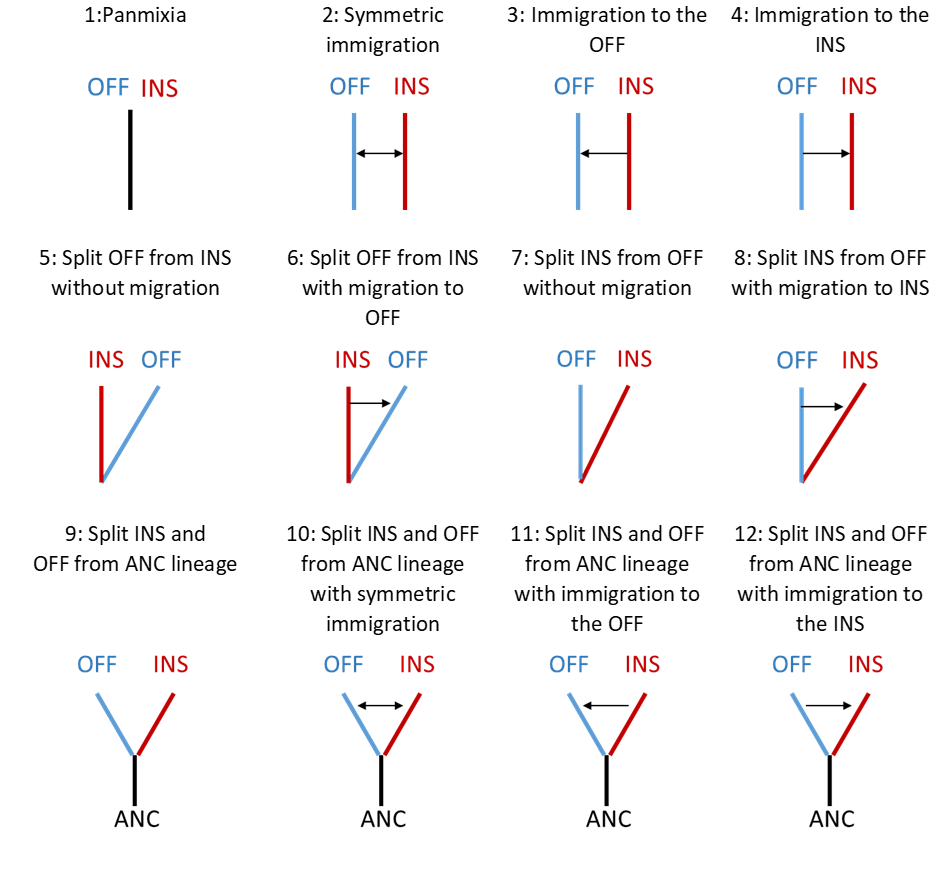 |
| --- |
| **Figure S1.1**. Twelve models specifying different population histories were tested using Migrate-n software to evaluate the different models based on their marginal likelihood. OFF, offshore lineage; INS, inshore lineage; ANC, ancestral lineage. |

| 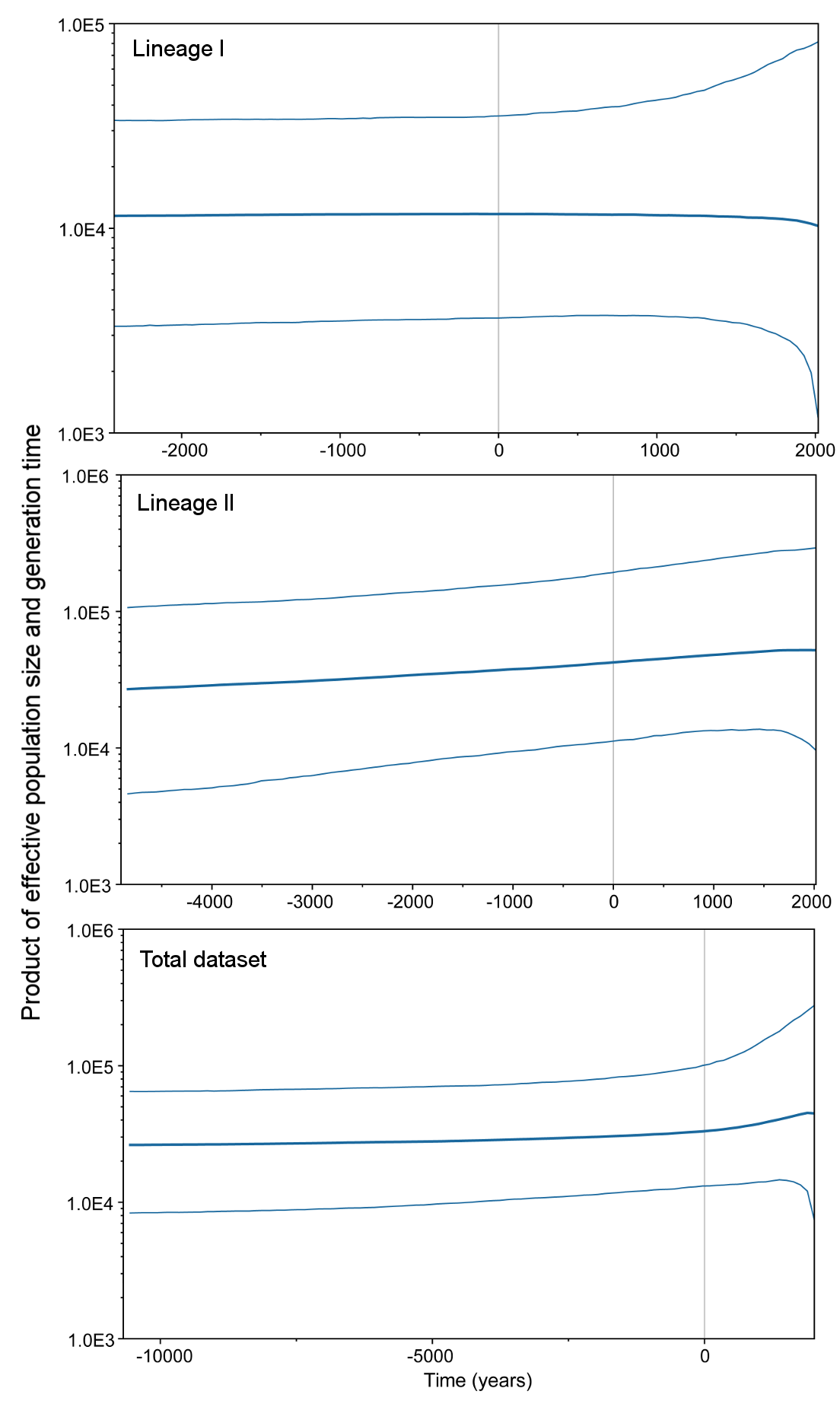 |
| --- |
| **Figure S1.2**. Bayesian skyline plot (BSP) showing population size dynamic for *N. norvegicus* haplotypes from lineage I, lineage II and total dataset. The y-axis indicates effective population size scaled by mutation rate as a function of time. The dark blue line shows the median BSP estimate while the light blue lines show the upper and lower 95% highest posterior density limits. |

| 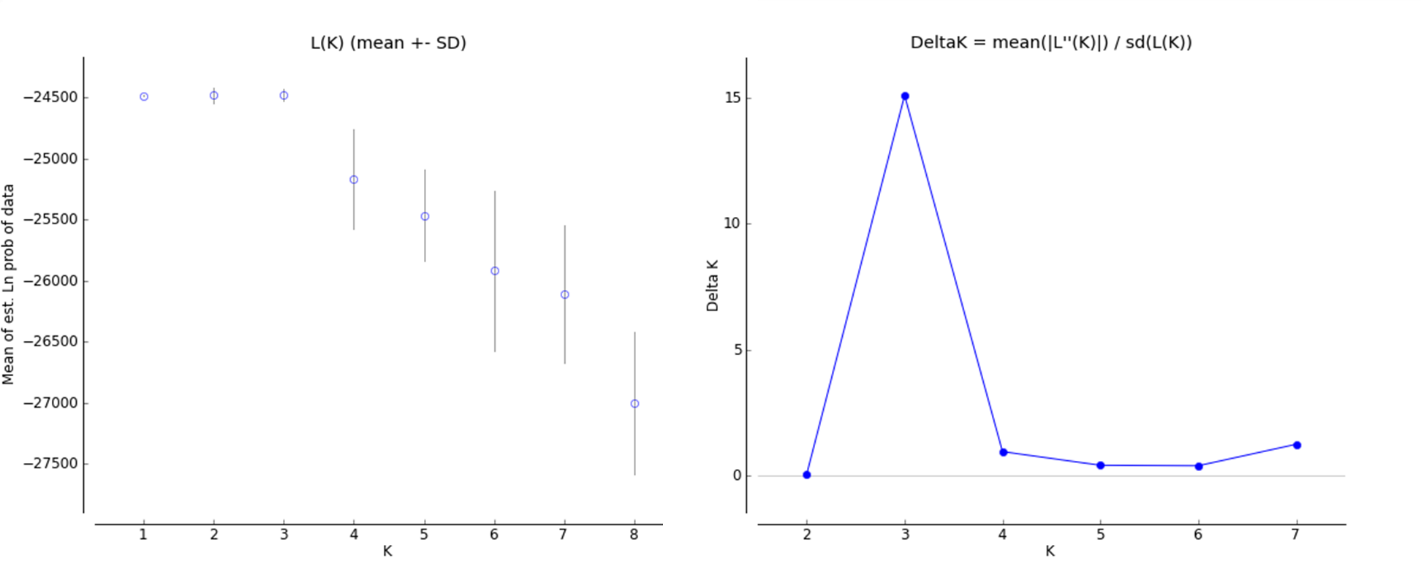 |
| --- |
| **Figure S1.3.** Plots of mean likelihood values of each K and variance per K value (left) and Delta K (ΔK) values given by STRUCTURE Harvester analysis of 482 Norway lobster *Nephrops norvegicus* samples using 10 polymorphic microsatellite markers (right). |

| 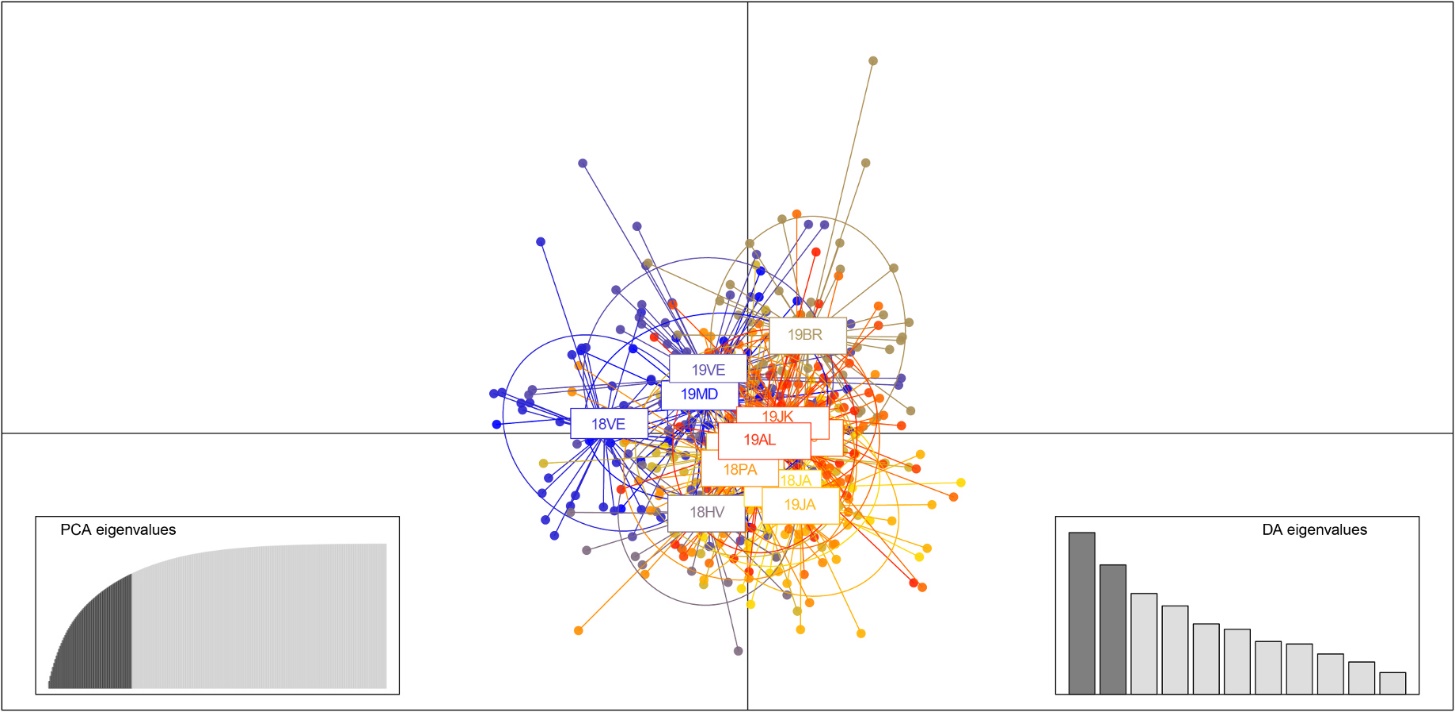 |
| --- |
| 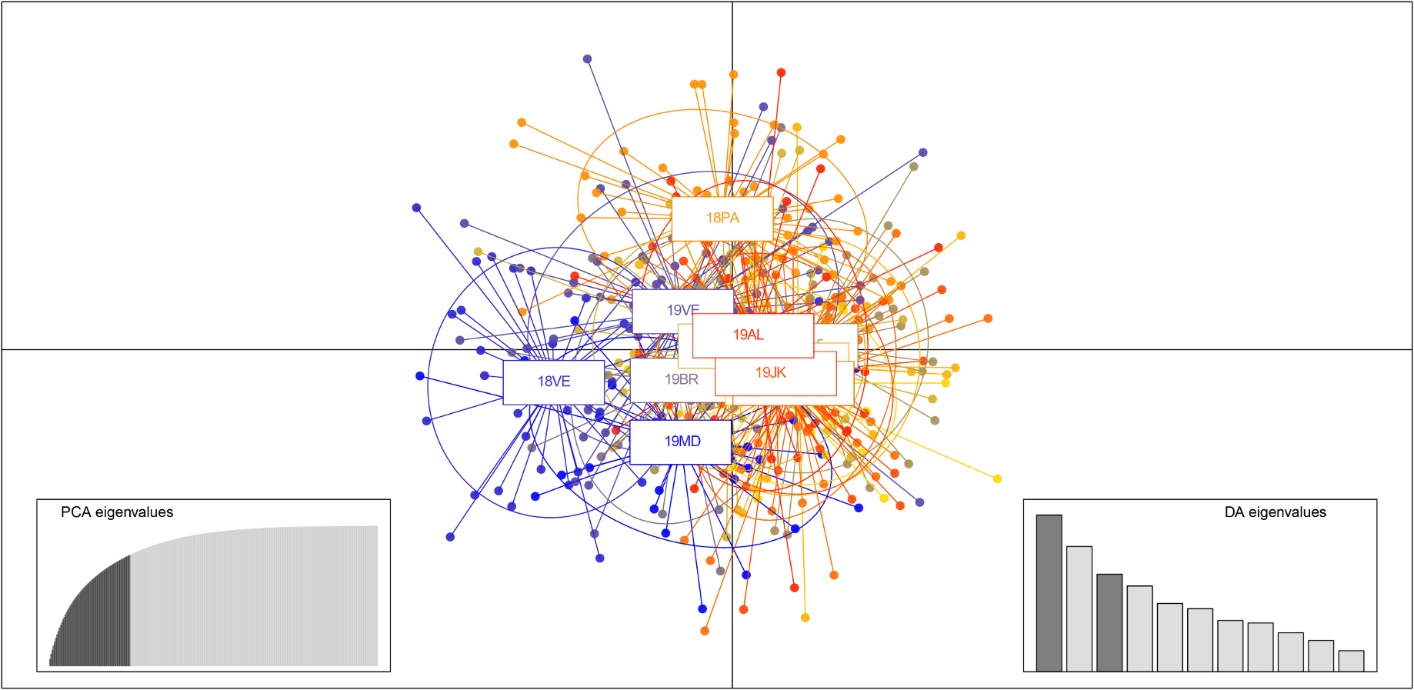 |
| **Fig. S1.4.** Scatterplots of the Discriminant Analysis of Principal Components (DAPC) model with sampling locality as a prior. Points are individual genotypes of 482 Norway lobster *Nephrops norvegicus.* The first plot represents the amount of genetic variation captured by the first two discriminant factors (DA1-DA2) while the second illustrates the variation by the first (x-axis) and third (y-axis) DA factor (D1-D3). |

**References**

Bracken-Grissom, H. D., Ahyong, S. T., Wilkinson, R. D., Feldmann, R. M., Schweitzer, C. E., Breinholt, J. W., ... & Crandall, K. A. (2014). The emergence of lobsters: phylogenetic relationships, morphological evolution and divergence time comparisons of an ancient group (Decapoda: Achelata, Astacidea, Glypheidea, Polychelida). *Systematic Biology*, *63*(4), 457-479.

Drummond, A. J., Ho, S. W. Y., Rawlence, N., & Rambaut, A. (2007). A rough guide to BEAST 1.4. Available at: http. *beast-mcmc. googlecode. com/files/BEAST14 Manual 6July2007*.

Posada D, Crandall KA (1998) MODELTEST: testing the model of DNA substitution. Bioinformatics 14:817–818

Rambaut A (2009). FigTree, a graphical viewer of phylogenetic trees. *Inst Evol Biol Univ Edinburgh*.

Rambaut A, Drummond AJ, Xie D, Baele G, Suchard MA (2018). Posterior summarization in Bayesian phylogenetics using Tracer 1.7. *Syst Biol* 67: 901–904.
